# Bidirectionally Enhanced Reaction Kinetics in Vanadium Redox Flow Battery via Regulating Mixed-Valence States in Perovskite Electrodes

**DOI:** 10.1007/s40820-025-02060-0

**Published:** 2026-02-03

**Authors:** Yingqiao Jiang, Ming Li, Jiaye Ye, Lei Dai, Haoran Jiang, Ling Wang, Zhangxing He

**Affiliations:** 1 School of Chemical Engineering, North China University of Science and Technology, Tangshan, 063009 People’s Republic of China; 2 School of Chemistry and Physics, Faculty of Science, Queensland University of Technology, Brisbane, QLD 4000 Australia; 3 Department of Energy and Power Engineering, Tianjin University, Tianjin, 300072 People’s Republic of China

**Keywords:** Vanadium redox flow battery, Perovskite, Selective regulation, Electronic structure, Reaction kinetics

## Abstract

Various metal oxide catalysts have been utilized to enhance the electrode reaction kinetics in vanadium redox flow battery (VRFB). However, the determining factor governing their catalysis is still insufficiently understood. Herein, selectively doping of Sr and Ce at La site of LaMnO_3_ perovskite (LSMO and LCMO) was used to modulate chemical environments of Mn ion activity donors, thereby boosting vanadium redox reaction processes. Sr doping increases the valence state of Mn ions, making it easier for Mn ions to take an electron from the electrode and transfer it to V^3+^ ions, which lowers the reaction energy barrier of V^3+^/V^2+^ redox processes. Conversely, Ce doping decreases the Mn valence and increases the oxygen vacancies, boosting the charge transfer and mass transfer of VO^2+^/VO_2_^+^ redox processes. Theoretical calculation further demonstrates that doping Sr and Ce enhances the vanadium ion’s ability for charge transfer and adsorption. Compared with pristine VRFB, the VRFB with LSMO- and LCMO-modified anode and cathode, respectively, exhibits an excellent energy efficiency (EE) of 67% at a high current density of 300 mA cm^−2^ and an increased EE of 15% at 150 mA cm^−2^. This study is critical for promoting fundamental understanding and offering a design strategy for achieving superior-performance metal-based electrocatalysts in VRFB.

**Supplementary Information:**

The online version contains supplementary material available at 10.1007/s40820-025-02060-0.

## Highlights

- A selectively regulating strategy for chemical environments of Mn ion activity donors in LaMnO_3_ perovskite can bidirectionally enhance vanadium reaction kinetics.
- The key reactive sites and control steps of perovskite on vanadium redox reactions are established based on electrochemical tests and theoretical calculation.
- Sr and Ce doped LaMnO_3_ as anode and cathode catalysts of the vanadium redox flow battery (VRFB) , respectively, synergistically improves the VRFB’s energy storage performance.

**Supplementary Information:**

The online version contains supplementary material available at 10.1007/s40820-025-02060-0.

## Graphical abstract

**Figure:**
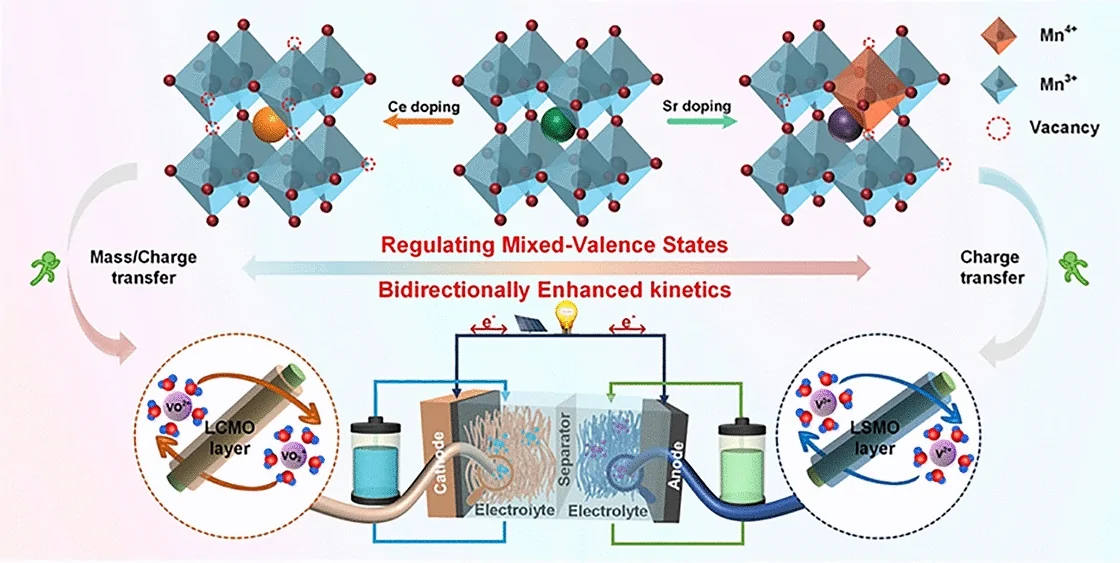

## Introduction

The growing severity of the climate crisis calls for urgent global efforts on carbon reduction. In response, the optimization of the energy structure has emerged as a practical and effective national strategy. The renewable resources of wind and solar power have been rapidly developed, driving the advancement of various energy conversion technologies such as photovoltaic power generation technology, wind power generation technology, and geothermal power generation technology. However, the inherent intermittent and fluctuating nature of renewable energy, compounded by its compatibility challenges with the grid, constitutes the primary technical bottleneck in the energy conversion process. Long-duration energy storage technologies, through the cross-period storage and release of energy alongside optimized allocation, provide a viable technical pathway to overcome this bottleneck [1–6]. Among various energy storage technologies, vanadium redox flow battery (VRFB) is a highly acclaimed large-scale energy storage system owing to its brilliant features, including decoupled capacity and power, high efficiency, long lifespan, and high safety [7–9].

In VRFB, the redox pairs, V^3+^/V^2+^ and VO^2+^/VO_2_^+^, are employed as the active species and take place redox reactions on the surface of electrodes as the following equations:

1 $${\mathrm{Anode}}:{\mathrm{V}}^{3 + } + e^{ - } \rightleftharpoons {\mathrm{V}}^{2 + }$$

2 $${\mathrm{Cathode}}:{\mathrm{VO}}^{2 + } + {\mathrm{H}}_{2} {\mathrm{O}} \rightleftharpoons {\mathrm{VO}}_{2}^{ + } + 2{\mathrm{H}}^{ + } + e^{ - }$$

3 $${\text{Full cell}}:{\mathrm{VO}}^{{2 + }} {\text{ + H}}_{{2}} {\text{O + V}}^{{3 + }} { } \rightleftharpoons {\text{ VO}}_{{2}}^{ + } {\text{ + 2H}}^{ + } {\text{ + V}}^{{2 + }}$$

The anodic reaction involves one electron transfer, while the cathodic reaction involves one electron, protons transfer and band breaking. In addition, both anodic and cathodic reactions generally include several steps: vanadium ion diffusion and adsorption/desorption, charge transfer, and electron transport [10–12]. Based on the cell principles, it is evident that the overall performance of VRFB is comprehensively influenced by the synergistic interactions among three key components of the electrolyte, membrane, and electrode [13]. Specifically, the active materials of vanadium ions are stored within the electrolyte, with the VRFB’s energy density primarily determined by vanadium ion concentration. However, its solubility and thermal stability are constrained by temperature and concentration conditions. Consequently, research efforts have centered on developing hydrochloric acid mixed electrolytes such as HCl-/H_2_SO_4_-supported electrolyte, and functional additives including inorganic stabilizers, polyols, and surfactants [14–16]. Moreover, it is the membrane that serves to separate the anode and cathode electrolytes, preventing cross-contamination of ions, while simultaneously ensuring efficient H^+^ ion transport to maintain the system’s charge equilibrium. The common membranes are Nafion series due to its exceptional proton conductivity and chemical stability. However, these membranes are high cost and difficult in balancing ion selectivity and conductivity. To address this challenge, researchers are actively developing novel membrane materials, encompassing non-fluorinated porous polymer membranes, organic–inorganic composite membranes, and multi-layered membranes with gradient structures [17–19]. These aim to achieve rapid proton conduction with high ion selectivity and decrease the cost. Last but not least, the electrode takes place for vanadium redox reactions and therefore directly dictates the power output of VRFB. Currently, carbon-based materials like graphite felt are widely employed as electrodes of VRFB [20–22]. These electrodes exhibit high conductivity, excellent chemical stability, and 3D porous structure, which provide a fast electron and electrolyte transport path. However, insufficient active site and weak hydrophilicity of carbon electrode hinder the diffusion and charge transfer of vanadium ion during cell operation. To resolve the issues, numerous methods have been used to modify the carbon electrode such as by introducing hydrophilic groups, heteroatoms, and high-activity catalysts [23–36]. In contrast with the significant expense of developing new electrolytes or membranes, electrode modification offers a relatively low-cost approach to enhancing battery performance.

Introducing catalyst gets a wide attention due to their high activity, abundant species, and design flexibility. Among various catalysts, metal oxide catalysts, such as Mn_3_O_4_, TiO_2_, ZrO_2_, etc., are quite preferred for modification of carbon electrodes due to low cost, high activity, large tunable space, and diverse species [37, 38]. The electrocatalytic activity of metal oxide primarily originates from the d-electron orbitals of transition metal cation and their variable valence states, which determine the charge transfer and adsorption behavior of reaction intermediates. To further enhance catalytic performance, lattice engineering strategies such as metal/non-metal doping, hydrogen reduction, and protonation have been investigated [39, 40]. These approaches strengthen the intrinsic activity and surface density of active sites, leading to an improvement in redox reaction kinetics. Recent research has increasingly focused on composite metal oxides, which exhibit exceptional performance through synergistic effects between different metal cations. Materials such as NiCo_2_O_4_ and CoMoO_4_, perovskites, and high-entropy metal oxides all demonstrate significantly higher active site densities than single-metal oxides [41]. This strategic evolution toward complex metal oxides is emerging as a key direction for developing high-performance electrodes. The perovskite-type metal oxides with general formula of ABO_3_, such as LaMnO_3_, SrZrO_3_, LaNiO_3_, etc., have been demonstrated to have excellent catalytic effect on the redox reaction, where B ions mainly determine its electrochemical activity [42–45]. However, to our best knowledge, there are no reports that provide an in-depth analysis of the mechanism of how the valence states of B in ABO_3_ dominates its electrochemical activity for the redox reaction of VO^2+^/VO_2_^+^ and V^3+^/V^2+^. This has significant reference for the design and construction of high-performance electrodes and catalysts based on perovskite-type metal oxides with similar structures.

To further reveal the mechanism of the chemical environment of B-site elements as core active center of ABO_3_-type perovskite materials, it is feasible to regulate the chemical environment at the B site by doping the A site of perovskite, without introducing other B-site elements [46]. As a classic ABO₃-type perovskite, LaMnO_3_ possesses both excellent structural regulation flexibility and chemical stability. It’s A site exhibits high doping tolerance, enabling compatibility with doping elements of different valences and ionic radii without damaging the crystal framework. Additionally, the valence state of B-site Mn can be reversibly adjusted through external regulation, and the generation and migration of oxygen vacancies also have good adjustability [47]. Therefore, this study employs a rational doping strategy on LaMnO_3_, selecting Sr^2+^ and Ce^4+^ as A-site dopants to regulate the Mn chemical state, and further employs them to catalyze the redox reactions of VO^2+^/VO_2_^+^ and V^3+^/V^2+^ bidirectionally. The design leverages the synergistic effects of ionic radius mismatch and valence differences to concurrently regulate the Mn^3+^/Mn^4+^ ratio and oxygen vacancy concentration. Thereby, Sr doping is intended to enhance V^3+^/V^2+^ reaction kinetics, while Ce doping targets the VO^2+^/VO_2_^+^couple. The determined factors of reaction process of both redox pairs are confirmed by the combination of experimental results and first principle. Finally, the Sr- and Ce-doped LaMnO_3_ is applied as the anode and cathode catalysts for VRFB, respectively, which significantly improves the VRFB’s performance by enhancing the diffusion, adsorption, and charge transfer of the redox pairs. This work employs a strategy of separate Sr and Ce doping in LaMnO_3_ to independently construct active centers suitable for the V^3+^/V^2+^ and VO^2+^/VO_2_^+^ redox couples, respectively. The study demonstrates that targeted modulation of the local chemical environment through elemental doping provides an effective approach for designing composite metal oxide catalysts of regulable metal ions with specific electrochemical functions.

## Experimental Section

### Preparation of Materials

All A.R.-grade chemicals were directly utilized. Sr- and Ce-doped lanthanum manganate perovskite powders were prepared by sol–gel method. La(NO_3_)_3_·6H_2_O (99%, Macklin), Mn (NO_3_)_2_·4H_2_O (98%, Macklin), Sr (NO_3_)_2_ (99.5%, Damao chemical reagent factory), Ce (NO_3_)_3_·6H_2_O (99.5%, Aladdin) were used as metal ion precursors, respectively. Firstly, stoichiometric metal nitrates, citric acid (99.5%, Aladdin), and ethylenediaminetetraacetic acid (99.5%, Aladdin) were added to deionized water. In continuous stirring, ammonia water (28%, Aladdin) was added drop by drop to form a transparent solution. The pH was controlled between 8 and 9. The molar ratio of citric acid: ethylenediaminetetraacetic acid: total metal ions was 1.5:1:1. Then, the solution slowly evaporated water at 80 °C to form a gel, and the gel was dried in an oven to form a loose hardened xerogel, followed by a calcination at 200 °C in air for 6 h. And then, the obtained black powder was calcined at 900 °C in air for 3 h to form the final sample. The obtained samples with Sr doping molar ratio at 10%, 20%, 40%, and 80% were named after LSMO-X (*X* = 10, 20, 40, and 80), respectively, while the samples with Ce doping molar ratio at 5%, 10%, and 15% were named after LCMO-Y (*Y* = 05, 10, and 15), respectively. The undoped LaMnO_3_ sample was referred to as LMO.

### Characterization Analysis

The crystal phase of samples was detected by the *X*-ray diffraction instrument (XRD, D8 Advance A25, Bruker). The morphology, element distribution, and electronic structure of samples were observed by scanning electron microscope (SEM, JSM-IT100) and transmission electron microscope (TEM, JEOL-JEM-F200). The surface chemical state and structure defect of samples were studied by the *X*-ray photoelectron spectroscopy (XPS, the *K*-Alpha 1063) with Al-Kα radiation and Raman spectroscopy (DXR 03040404), respectively. The wettability of the samples was evaluated by measuring the contact angle with distilled water, using a HARKE-SPCA instrument (Beijing Hake Test Instrument Factory).

### Electrochemical Test

The electrochemical activity of samples was studied by using the CHI660E and AutoLab PGSTAT302N electrochemical workstations equipped with cyclic voltammetry (CV) and electrochemical impedance spectroscopy (EIS) modules. In this testing process, the saturated calomel electrode (SCE) was the reference electrode, a platinum sheet was the counter electrode, and the glassy carbon electrode or graphite felt was the working electrode, respectively. When the glassy carbon electrode (GCE) served as the working electrode, the following was how it was made: 5 mg catalyst and 5 mg conductive carbon black (Super P, SP) were added in 10 mL N, N-dimethylformamide (DMF) and treated with ultrasonic for 3 h to obtain the catalyst ink. 10 μL ink was added onto the GCE drop by drop. The electrochemical tests were carried out in 3.0 M H_2_SO_4_ solutions comprising 1.6 M V^3+^ for V^3+^/V^2+^ reaction and 1.6 M VO^2+^ for VO^2+^/VO_2_^+^ reaction, respectively. The EIS was tested in frequency range of 10^−1^ − 10^5^ Hz. The graphite felt with perovskite modification, as the working electrode, was prepared as the follows: Firstly, 10 mg perovskite was first added in 10 mL DMF. After 3-h ultrasonic dispersion, the catalyst ink was obtained and the original graphite felt was soaked in the ink to absorb it. Then, the graphite felt was fully dried at 80 °C. The steps were repeated until the ink was adsorbed fully by the graphite felt. The electrochemical tests were executed in electrolyte of 0.1 M V^3+^  + 3.0 M H_2_SO_4_ and 0.1 M VO^2+^  + 3.0 M H_2_SO_4_ solutions for V^3+^/V^2+^ and VO^2+^/VO_2_^+^ reactions, respectively. The EIS was measured in frequency range of 1 − 10^5^ Hz.

### Calculation Methods

All computations were conducted on the basis of the density functional theory by using the Vienna ab initio simulation package (VASP). The exchange–correlation functional adopted the generalized gradient approximation (GGA) in the form of Becke86. The optB86b-vdW functional was used to optimize structure for the weak interlayer van der Waals interactions. Energy cutoff for the plane-wave expansion was set to 500 eV. The convergence threshold was set as 10^−5^ eV in energy for structure optimization. A vacuum layer greater than 15 Å was taken to avoid the interaction between adjacent images. A supercell of four-layer slab was used to represent the absorbed surface, and the top two atom layers were relaxed. For computing the charge density difference of LaMnO_3_ slabs, the 2D Brillouin zone integration using the Γ-center scheme was sampled with a 14 × 14 × 1 grid.

### Charge and Discharge Test

The energy storage performance of VRFB was tested on the equipment (CT2001A, Wuhan LAND Electronic Co. Ltd.). LSMO-20 and LCMO-10 modified graphite felts were, respectively, used as the anode and cathode to assemble the modified VRFB as LSMO/LCMO VRFB. For comparison, pristine graphite felts served as the VRFB’s anode and cathode. The Nepem-1110 ion-change membrane was employed as the membrane between the cathode and anode. The catholyte and anolyte were 0.8 M V^3+^  + 0.8 M VO^2+^  + 3.0 M H_2_SO_4_. 25 mL of electrolyte was circulated throughout the VRFB by two peristaltic pumps at two half-cells, respectively. The battery’s polarization curve was tested by first charging it to 1.65 V and then discharging it for 300 s at each current density.

## Results and Discussion

**Fig. 1:**
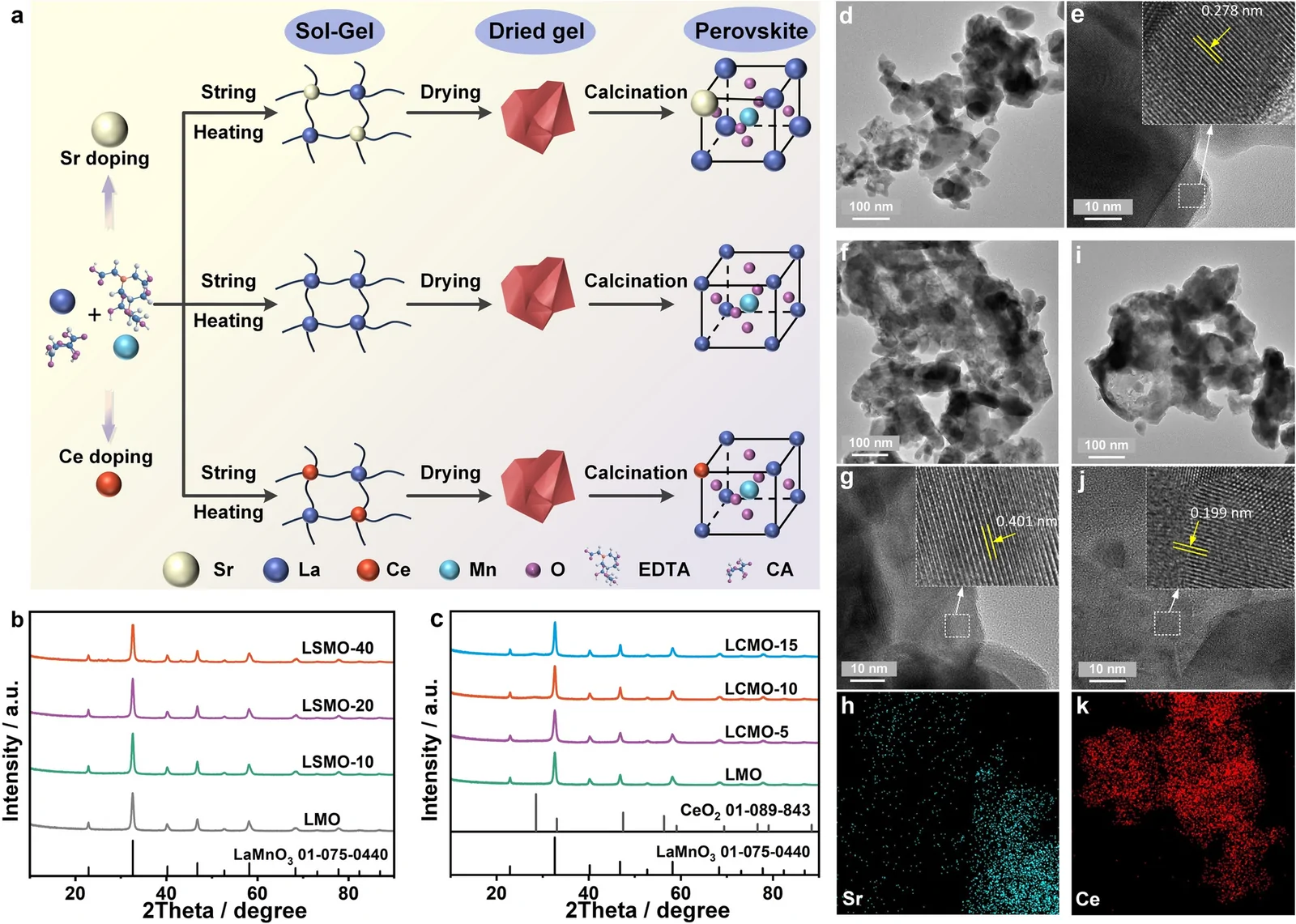
**a** Preparation process of perovskites. XRD patterns of **b** Sr-doped LaMnO_3_ and **c** Ce-doped LaMnO_3_. **d** TEM and **e** HRTEM images of LMO. **f** TEM, **g** HRTEM, **h** Sr element EDS mapping images of LSMO-20. **i** TEM, **j** HRTEM, **k** Ce element EDS mapping images of LCMO-10

Figure 1a shows the sol–gel preparation processes of perovskite, where Sr and Ce with different ionic radius and charge numbers are doped in crystal lattice to replace La^3+^ of LaMnO_3_ respectively. The XRD pattern of LMO sample in Fig. 1b exhibits the well-distinct and high-intensitive diffraction peak, exactly matching the standard card of cubic LaMnO_3_ perovskite (JCPDS 01–075-0440). This indicates that the well-crystallized pure phase of LaMnO_3_ perovskite is obtained. The XRD patterns of LSMO samples are almost the same as that of LMO when the ratio of doped Sr is not exceeding 40%. However, when the doping ratio of Sr reaches 80%, some impurity peaks appear corresponding to SrCO_3_ (Fig. S1). For LCMO samples **(**Fig. 1c**)**, when the Ce doping ratio is under less than 15%, no impurity peak appears. But after that, a miscellaneous peak appears at 28.2°, corresponding to CeO_2_ [48, 49]. These results can be attributed to the ionic radius of Sr^2+^ (1.18 Å) > La^3+^ (1.03 Å) > Ce^4+^ (0.97 Å), and thus, the LaMnO_3_ lattice possesses a greater capacity for Sr^2+^ than Ce^4+^.

SEM and TEM tests were used to observe morphology and electronic structure of perovskites. As shown in Fig. S2a, b, LMO is composed of 50-nm nanoparticles. Sr- and Ce-doped samples exhibit the similar morphology compared with LMO suggesting that doping Sr (10% ~ 40%) and Ce (5% ~ 15%) make no remarkable influence on the morphology (Figs. S3 and S4, 1d, f, i). It is noteworthy that Sr and Ce doping increases the lattice spacing to 0.401 nm and decreases it to 0.199 nm from 0.278 nm, respectively, owing to the difference in ionic radii between Sr and Ce compared to La (Fig. 1g, j). Moreover, as shown in the EDS mapping results (Figs. 1h, k and S4, S5), the doped elements, Sr and Ce, are uniformly distributed in LSMO-20 and LCMO-10, respectively.

**Fig. 2:**
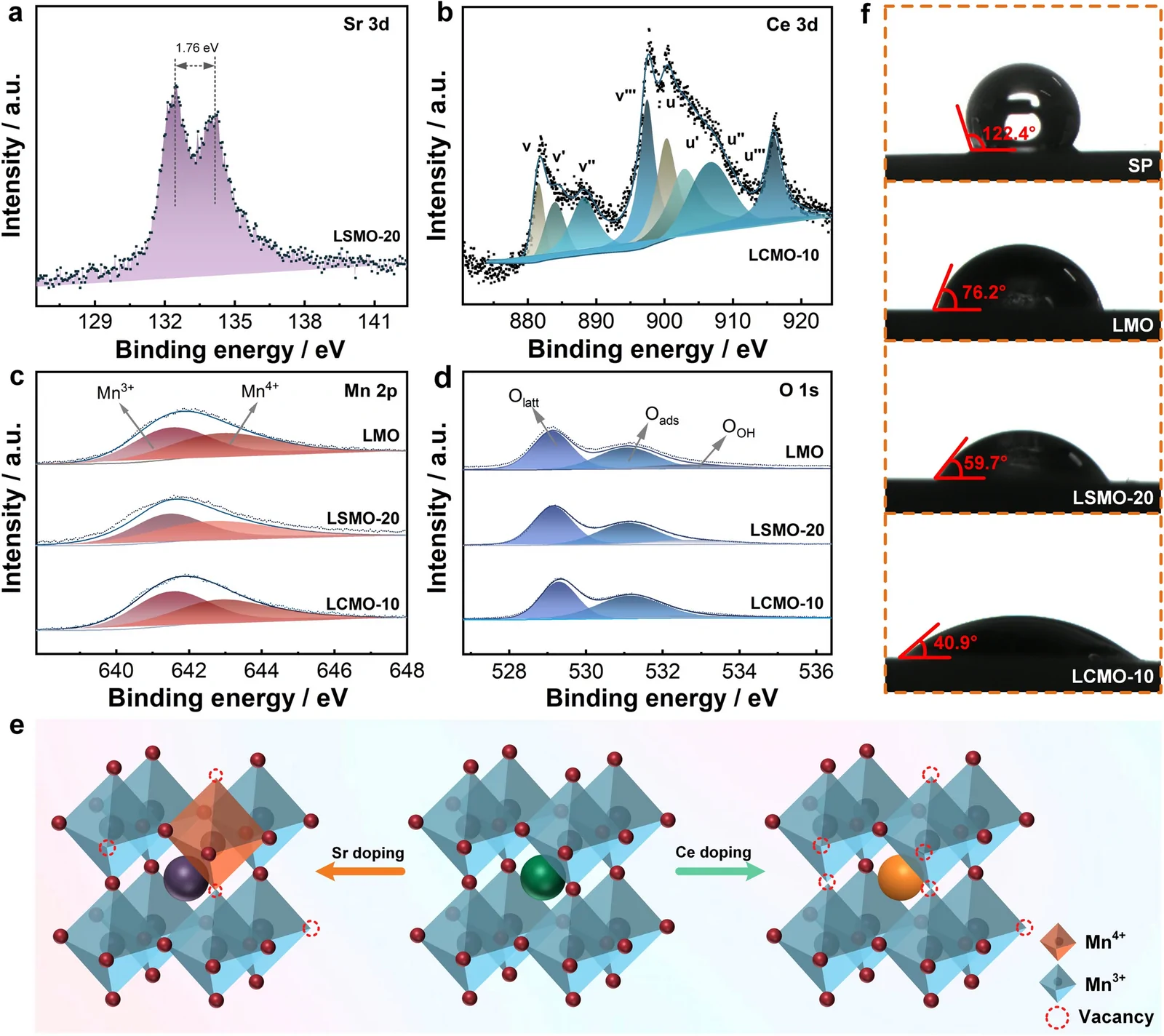
High-resolution XPS spectra of **a** Sr 3*d*, **b** Ce 3*d*, **c** Mn 2*p*, and **d** O 1*s* of samples. **e** Schematic diagram of Sr and Ce doping LaMnO_3_. **f** Contact angles of SP, LMO, LSMO-20, and LCMO-10

XPS was used to study the chemical composition of these perovskites. From Figs. S6 and S7, LMO contains La, Mn, O, and C elements, while LSMO and LCMO samples contain additional Sr and Ce elements, respectively. The chemical environment in which this constituent element exists is analyzed by the high-resolution Sr 3*d*, Ce 3*d*, O 1*s*, and Mn 2*p* spectra. In Figs. 2a and S8, the Sr 3*d* spectra include two splitting peaks, corresponding to the binding energies of Sr 3*d*_3/2_ (134.15 eV) and Sr 3*d*_5/2_ (132.39 eV), respectively, with the spacing of the two peaks of 1.76 eV. It suggests Sr^2+^ doped into LaMnO_3_ lattice. Moreover, Figs. 2b and S9 show the Ce 3*d* spectra exhibit more than 10 peaks, which correspond to various oxidation states and multi-electron splitting interactions. These peaks are classified into two major parts at 881 −897 and 898 −917 eV, matching spin–orbit splitting of 3*d*_3/2_ and 3*d*_5/2_, respectively. Among these peaks, v′ and u′ correspond to Ce^3+^, and the other peaks correspond to Ce^4+^, which suggests that cerium coexists in LaMnO_3_ lattice in two ionic states of Ce^3+^ and Ce^4+^ [50]. In addition, Ce^4+^ is the dominated in LCMO samples, because of its good stability at high calcination temperature.

The effect of Sr and Ce doping on Mn and O chemical states is explored. In Mn 2*p* spectrum for LMO sample (Fig. 2c), an asymmetric Mn 2*p*_3/2_ peak is observed at 642.0 eV, which is decomposed into two peaks of 641.5 eV for Mn^3+^ and 642.8 eV for Mn^4+^, respectively. This indicates that Mn^4+^ and Mn^3+^ coexist in LaMnO_3_ perovskite lattice, with the Mn^3+^/Mn^4+^ ratio of 1.08. When Sr^2+^ is doped into LaMnO_3_ lattice to replace the La^3+^, the valence compensation and a larger ionic radius of Sr^2+^ result in the transition of Mn^3+^ to Mn^4+^ [51]. The concentration of Mn^4+^ initially increases and then decreases with the increase in Sr doping ratio (Fig. S10a). The Mn^3+^/Mn^4+^ ratio of LSMO-20 is reduced to 0.97. While, in LCMO samples, Mn also exists in two forms of Mn^3+^ and Mn^4+^. Due to charge compensation mechanisms, Ce doping increases the proportion of Mn^3+^ and generates more oxygen vacancies in LaMnO_3_, with oxygen vacancies playing the dominant role (Fig. S10b). The Mn^3+^/Mn^4+^ ratio of LCMO-10 is increased to 1.10. The alteration of Mn ion state is crucial for its electrochemical catalytic performance, because metal ion of different valence states has varying redox ability.

As shown in Figs. 2d and S11, the O 1*s* spectra are decomposed into three peaks at 529.3 − 529.5, 531.0, and 532.5 − 532.7 eV, corresponding to the lattice oxygen (O_latt_), the adsorbed oxygen (O_ads_), and oxygen in water molecules (O_OH_). Specifically, the O_ads_ concentration increases in the order of LMO (35%) < LSMO-20 (39%) < LCMO-10 (49%). This suggests that the introduction of Sr and Ce into LaMnO_3_ causes lattice defects and increases oxygen vacancies. Additionally, due to Ce with a smaller ionic radius, it adopts a more complex mode of occupancy and generates a higher number of structural defects compared to Sr. The Raman spectra also demonstrate that Sr and Ce doping causes structure defect. At the same doping ratio, Ce introduces more defect than Sr (Figs. S12, S13). The effects of Sr and Ce doping on LaMnO_3_ lattice are summarized in Fig. 2e**.** Sr doping introduces Sr^2+^ ions at La^3+^ sites, creating electron holes that oxidize Mn^3+^ to Mn^4+^ for charge balance. In contrast, Ce doping incorporates Ce^4+^ ions at La^3+^ sites, providing excess electrons that reduce Mn^4+^ to Mn^3+^ while simultaneously increasing oxygen vacancy.

The metal–oxygen binding and oxygen vacancy have a huge effect on the wettability of perovskite samples, which was explored by contact angle test. As displayed in Figs. 2f and S14, the contact angles of perovskites are smaller compared to carbon materials such as Super P. Additionally, the contact angles of LSMO and LCMO samples are smaller than that of LMO. These findings suggest that doping Sr and Ce enhances the wettability of LaMnO_3_ perovskite. In addition, LCMO samples exhibit better wettability than LSMO samples, coinciding with the XPS analysis.

**Fig. 3:**
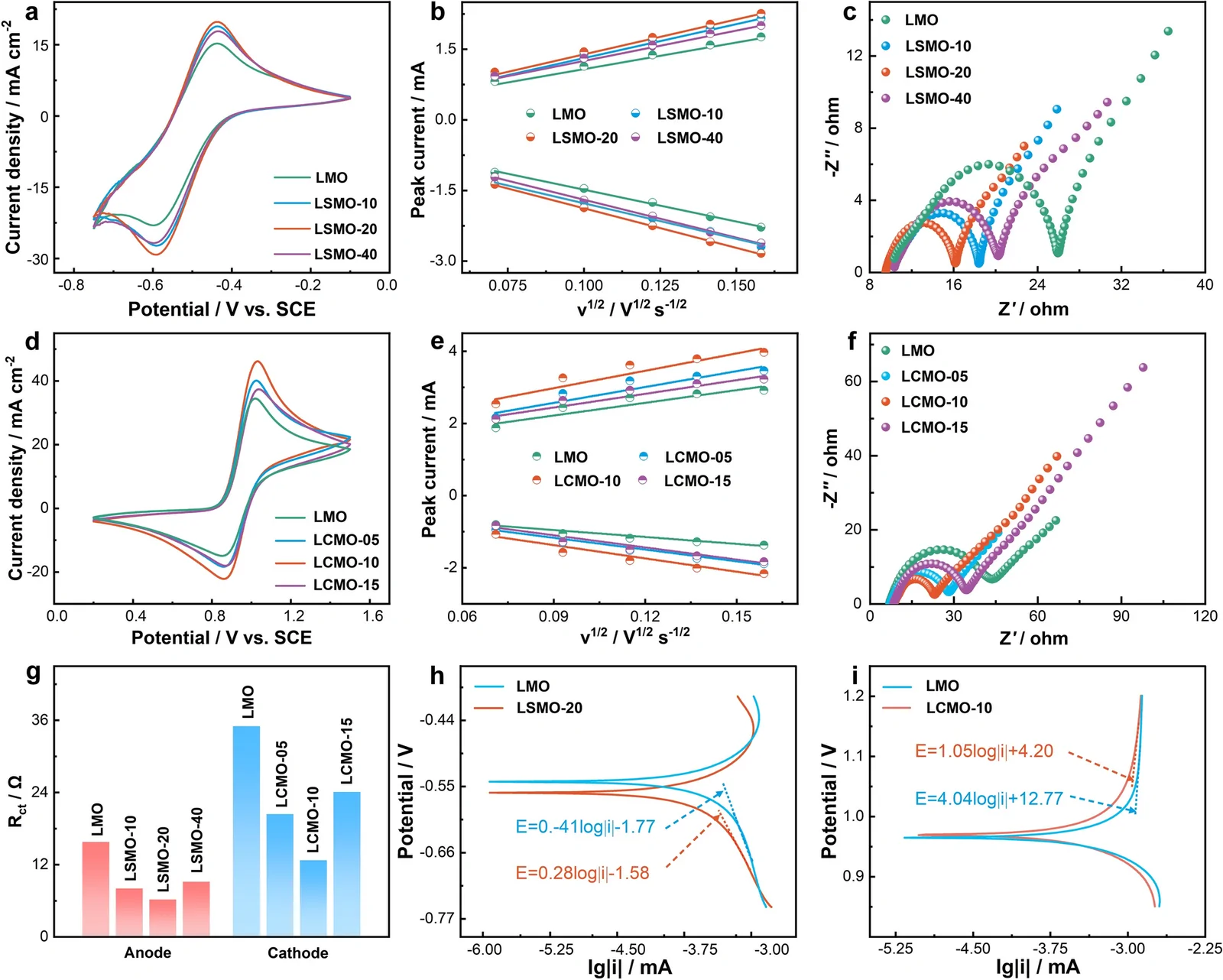
**a** CV curves at 10 mV s^−1^, **b** linear relation plots of peak current vs. square root of scan rates, **c** Nyquist plots at −0.45 V of LMO- and LSMO-modified GCE electrodes in 1.6 M V^3+^  + 3.0 M H_2_SO_4_ solution. **d** CV curves at 10 mV s^−1^, **e** linear relation plots of peak current vs. square root of scan rates, **f** Nyquist plots at 0.85 V of LMO- and LCMO-modified GCE electrodes in 1.6 M VO^2+^  + 3.0 M H_2_SO_4_ solution.** g** *R*_ct_ of different electrodes for anode and cathode reactions. Tafel plots for **h** V^3+^/V^2+^ and **i** VO^2+^/VO_2_^+^ reactions

The CV measurements were performed for exploring the electrochemical activity of LSMO for V^3+^/V^2+^ pair and LCMO for VO^2+^/VO_2_^+^ pair, respectively. As shown in Fig. 3a, d, under the same condition, LSMO and LCMO electrodes obviously enhance the electrochemical activity of V^3+^/V^2+^ and VO^2+^/VO_2_^+^ pairs, respectively. Among LSMO electrodes, due to the highest peak current density, LSMO-20 electrode (− 29.2 and 19.9 mA cm^−2^) displays the optimal electrochemical activity toward V^3+^/V^2+^ reaction. Similarly, LCMO-10 (peak current densities of 46.2 and − 22.1 mA cm^−2^) shows the best electrochemical kinetics for VO^2+^/VO_2_^+^ reaction than other LCMO electrodes. CV test at different scan rates was carried out for further investigating the electrochemical kinetics of vanadium redox reaction (Figs. S15, S16). According to the Randles–Sevcik equation, as displayed in Fig. 3b, e, the redox peak current is linearly related with the square root of the scan rate for each electrode. It means that V^3+^/V^2+^ and VO^2+^/VO_2_^+^ reactions are under control of the diffusion process [52]. Moreover, LSMO and LCMO obviously boost the diffusion of vanadium ions toward electrode compared with LMO.

As shown in Fig. 3c, f**,** in all Nyquist plots, semicircles dominate the high-frequency portion, while straight lines dominate the low-frequency sector. This result indicates that V^3+^/V^2+^and VO^2+^/VO_2_^+^ reactions are controlled by a combination of diffusion processes and charge transfer at the given polarization potential. The electrochemical parameters of each component fitted according to the equivalent circuit (Fig. S17) are listed in Tables S1 and S2. The ohmic resistances (*R*_s_) of LSMO and LCMO electrodes are slightly smaller than those of LMO electrodes, because a better hydrophilicity of LSMO and LCMO reduces the contact resistance of the electrode with the electrolyte. It is worth noting that the charge transfer resistances (*R*_ct_) of all LSMO and LCMO electrodes are significantly smaller than those of LMO electrodes (Fig. 3g). The results indicates that doping Sr and Ce boosts the charge transfer of V^3+^/V^2+^ and VO^2+^/VO_2_^+^ reactions, respectively. Moreover, the diffusion capacitance (*Q*_t_) and electric double-layer capacitance (*Q*_m_) of LSMO and LCMO electrodes are greater than those of LMO electrodes. This means that more vanadium ions diffuse to the electrode surface and constitute a larger electrical double-layer capacitor interface due to doping Sr and Ce.

Tafel plots were used to deeply investigate the electrocatalytic activities of Sr- and Ce-doped LaMnO_3_ for V^3+^/V^2+^ and VO^2+^/VO_2_^+^ reactions, respectively. As shown in Fig. 3h, i**,** the Tafel slopes of LSMO-20 (−0.28 V dec^−1^) for V^3+^/V^2+^ redox reaction and LCMO-10 (1.05 V dec^−1^) for VO^2+^/VO_2_^+^ redox reaction are lower than those of LMO (−0.41 V dec^−1^ and 4.04 V dec^−1^), which can be ascribed to more efficient active sites of LSMO-20 and LCMO-10.

**Fig. 4:**
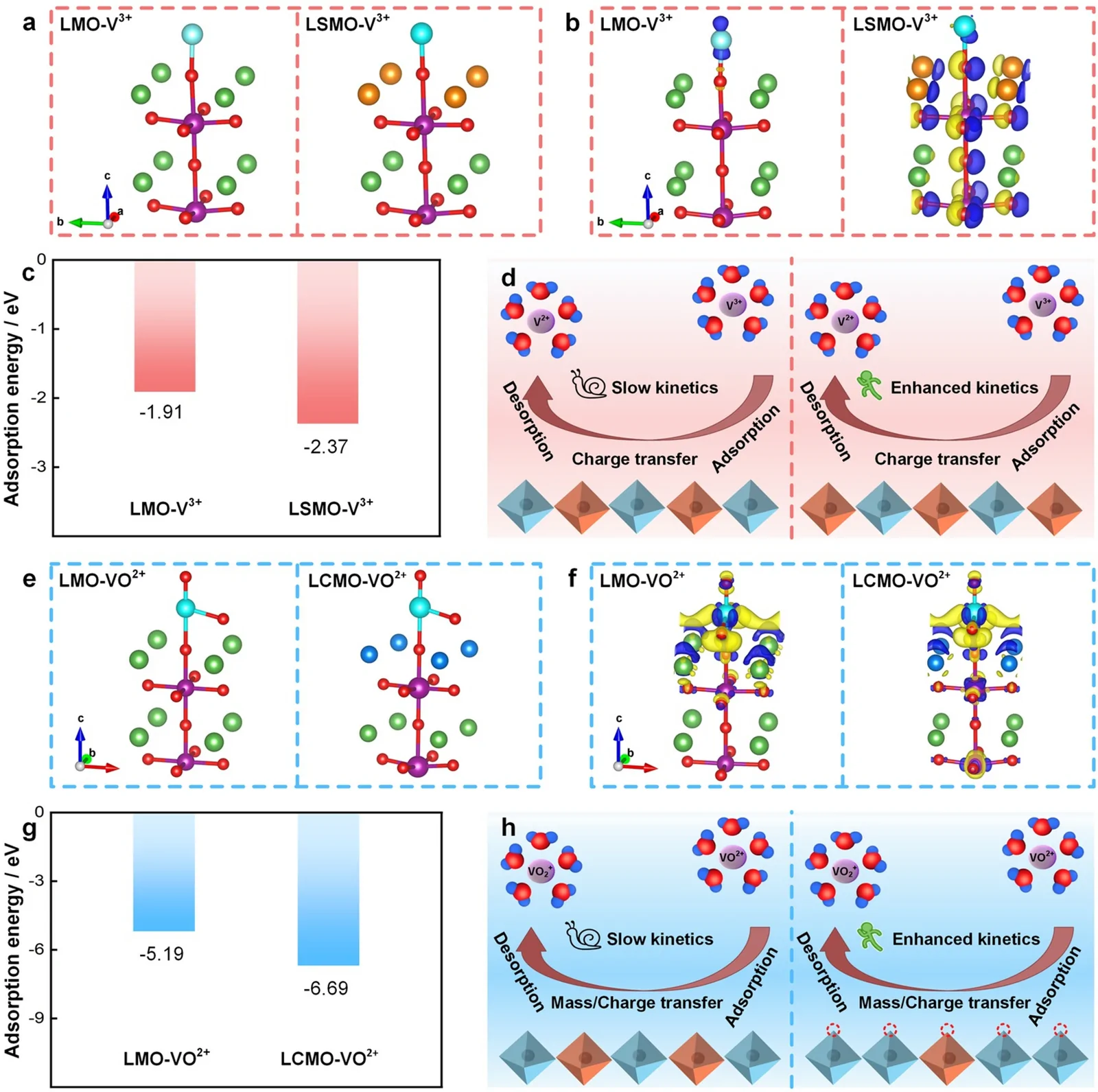
**a** V^3+^ adsorbed on LMO and LSMO. **b** Charge difference analysis for V^3+^-LMO and V^3+^-LSMO. **c** Comparison of adsorption energy for V^3+^-LMO and V^3+^-LSMO. **d** Schematic illustrations of V^3+^/V^2+^ reaction process. **e** VO^2+^ adsorbed on LMO and LCMO. **f** Charge difference analysis for VO^2+^-LMO and VO^2+^-LCMO. **g** Comparison of adsorption energy for VO^2+^-LMO and VO^2+^-LCMO. **h** Schematic illustrations of VO^2+^/VO_2_^+^ reaction process

Theoretical calculations were carried out to clarify the underlying the electronic structure of perovskite on the reaction processes of V^3+^/V^2+^ and VO^2+^/VO_2_^+^ couples at the atomic level. Firstly, we find from the adsorption model (Fig. 4a, e) that the vanadium ions are adsorbed on the O site of LaMnO_3_ perovskite. Specifically, in Fig. 4c, g, it is clear that the adsorption energy of vanadium ions on LSMO and LCMO is higher than that of the LMO, due to the Sr and Ce doping, respectively [53]. Furthermore, charge difference analysis (Figs. 4b, f and S18, S19) reveals that O atoms act as the reaction sites for vanadium ions, while the electronic states of Mn and O jointly impact charge transfer during vanadium redox reactions. Compared with LMO, Sr doping involves more charge in redox reaction and promotes charge transfer. V^3+^ ions preferentially adsorb onto oxygen vacancy and unsaturated coordination sites on the catalyst surface. The adsorbed V^3+^ interacts with neighboring Mn^3+^ active site, directly stripping their *e*_g_ orbital electrons to reduce to V^2+^. Concurrently, Mn^3+^ is oxidized to Mn^4+^. Subsequently, bulk electrons rapidly migrate to the surface via the Mn^3+^-O-Mn^4+^ double-exchange network, instantaneously reducing Mn^4+^ back to Mn^3+^ and completing active site regeneration. The mixed-valence state formed by Sr doping, coupled with the double-exchange mechanism, constitutes the core for achieving highly efficient and sustained catalysis. This finding is corroborated by a reduction in charge transfer resistance observed in EIS and an acceleration in reaction rates recorded in Tafel tests. For VO^2+^, we also find that doping Ce makes the Mn ion accept electrons more easily. Ce doping in LaMnO_3_ induces oxygen vacancies and locally reduces valence of Mn ion, jointly constructing a highly efficient catalytic interface. Within this interface, positively charged oxygen vacancies act as strong adsorption centers to capture and activate VO^2+^. The enhancement in hydrophilicity observed in contact angle measurement confirms the promotional effect of oxygen vacancy on interfacial mass transfer processes. The electron-rich environment of Mn^3+^-enriched zones provides a low-energy pathway for electron transfer from VO^2+^ to the catalyst, resulting in a low overpotential for its oxidation to VO_2_^+^. Concurrently, oxygen vacancies and their unsaturated sites promote water dissociation and proton exchange, synergistically completing the catalytic cycle of the entire oxidation reaction. Concurrent electrochemical testing reveals a decrease in charge transfer resistance and an accelerated Tafel reaction rate, collectively demonstrating that oxygen vacancies collaboratively improve adsorption and interfacial charge transfer processes. Briefly, the perovskite doping with Sr and Ce boosts the adsorption and charge transfer for V^3+^/V^2+^ and VO^2+^/VO_2_^+^, respectively, thus exhibiting faster reaction kinetics (Fig. 4d, h).

**Fig. 5:**
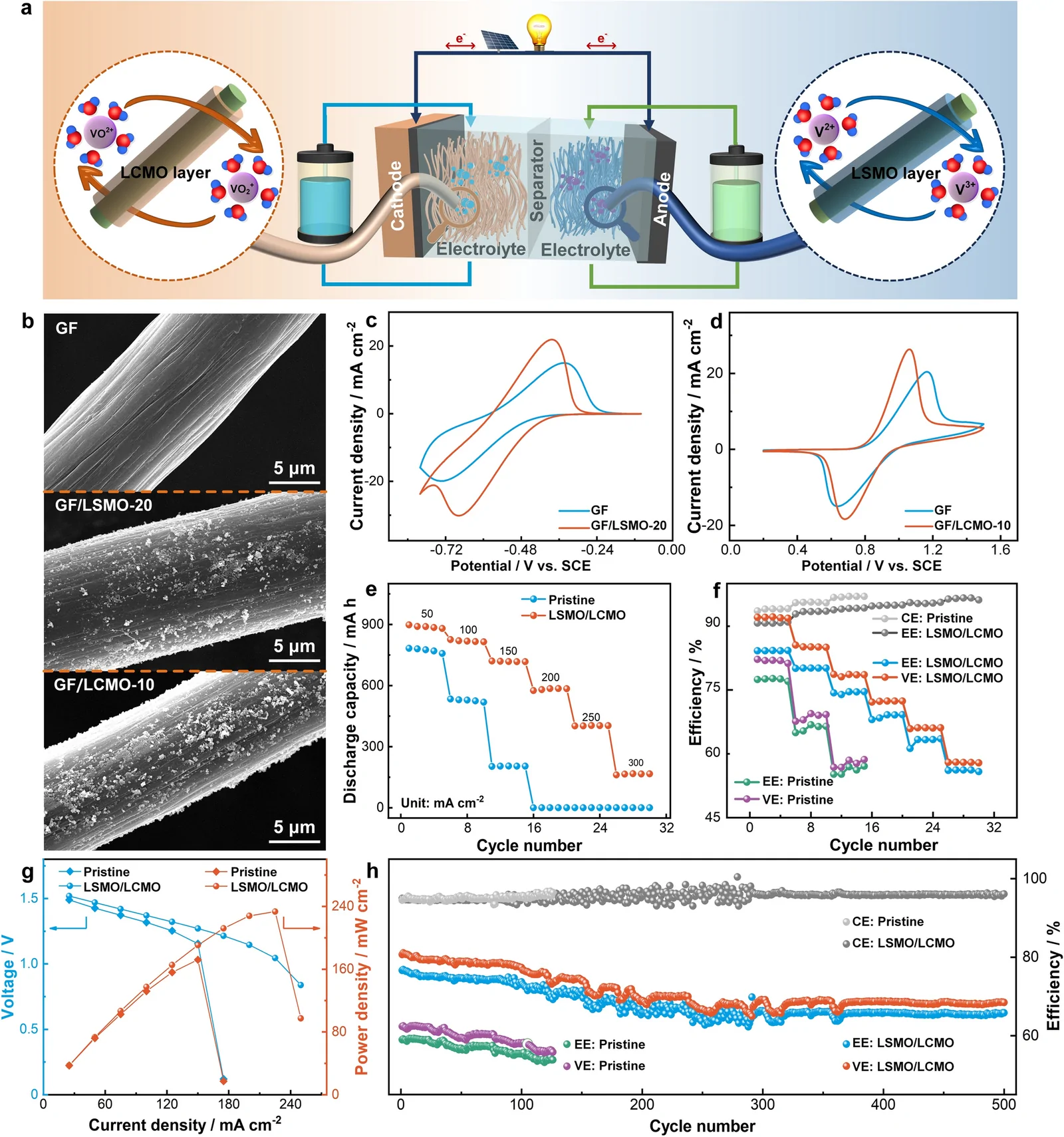
**a** Schematic illustration of VRFB with the LSMO-20 and LCMO-10 perovskite electrocatalysts. b SEM of GF, GF/LSMO-20 and GF/LCMO-10. CV curves of GF electrodes at 1 mV s^−1^ in **c** 0.1 M V^3+^  + 3.0 M H_2_SO_4_ and **d** 0.1 M VO^2+^  + 3.0 M H_2_SO_4_ solutions. **e** Discharge capacity, **f** efficiency and **g** polarization curves of the pristine VRFB and LSMO/LCMO VRFB at different current densities. **h** Efficiency of two VRFBs at 150 mA cm^−2^ for 500 cycles

Based on the above discussion, it can be seen that LSMO-20 and LCMO-10 have the highest reactivity enhancement for V^3+^/V^2+^ and VO^2+^/VO_2_^+^ reactions, separately. Therefore, the performance of the single cell would be evaluated next, and the modified electrodes GF/LSMO-20 and GF/LCMO-10 were employed as anode and cathode, respectively (Fig. 5a). Figure 5b demonstrates that LSMO-20 and LCMO-10 nanoparticles are uniformly distributed on GF, which greatly increase the reactive sites of electrode. Furthermore, the fundamental electrochemical activity of GF is explored by CV and EIS measurements. Compared with GF, GF/LSMO-20 and GF/LCMO-10 exhibit smaller peak potential differences and higher peak current densities, respectively, indicating that LSMO-20 and LCMO-10 improve the electrochemical kinetic and reversibility of GF for V^3+^/V^2+^ and VO^2+^/VO_2_^+^ reactions, respectively (Fig. 5c, d). Moreover, a lower *R*_s_ of both modified electrodes compared with GF (Fig. S20) suggests that LSMO-20 and LCMO-10 boost the charge transfer rate of GF electrode for V^3+^/V^2+^ and VO^2+^/VO_2_^+^ reactions.

Subsequently, the charge and discharge tests were used to assess the impact of GF/LSMO-20 and GF/LCMO-10 on single VRFB performance. As shown in Fig. 5e, LSMO/LCMO VRFB exhibits a higher discharge capacity at various current densities and keeps a slow discharge capacity decay compared with the pristine one. The hydrophilic LSMO-20 and LCMO-10 boost electrolyte utilization by facilitating vanadium ion adsorption and charge transfer, increasing the number of active ions in electrode reactions. Fig. 5f presents the current efficiency (CE), voltage efficiency (VE), and energy efficiency (EE) of cells. Among them, CE is used to evaluate the charge loss caused by self-discharge. LSMO/LCMO VRFB’s CE is slightly lower than that of pristine VRFB, because a higher discharge capacity of the LSMO/LCMO VRFB leads to a greater charge loss. VE is used for assessing the cell polarization consisting of ohmic polarization, concentration polarization, and electrochemical polarization. LSMO/LCMO VRFB presents a higher VE at all current densities compared to pristine VRFB. Especially at 150 mA cm^−2^, the cell with modified electrodes delivers a high VE of 78.6%, and its performance is much better than that of pristine one (58.4%). The improvement in VE is mainly due to the fact that LSMO/LCMO promotes the diffusion and charge transfer of vanadium ions, which greatly reduces the concentration and electrochemical polarizations of VRFB [54]. This is also confirmed by charge–discharge curves (Fig. S21), in which LSMO/LCMO VRFB exhibits a higher discharge voltage plateau and a lower voltage charge plateau than pristine VRFB at the same current density. Last but not least, EE is jointly affected by CE and VE (EE = CE × VE). LSMO/LCMO VRFB has excellent EE, especially at 150 mA cm^−2^, and its EE is increased by 17.3% compared with the pristine one. At a higher 300 mA cm^−2^, LSMO/LCMO VRFB even provides 66.5% EE, while pristine VRFB is out of work. To further evaluate the effects of LSMO/LCMO on polarization and power density of VRFB, the polarization curves of two VRFBs were tested. In Fig. 5g, LSMO/LCMO VRFB delivers a highest power of 233.3 mW cm^−2^ at 225 mA cm^−2^, while the pristine VRFB only reaches a highest power of 172 mW cm^−2^ at 150 mA cm^−2^. Meanwhile, LSMO/LCMO VRFB has a higher discharge voltage, indicating that the use of LSMO-20- and LCMO-10-modified electrodes reduces the polarization of VRFB.

In order to further explore the operational stability of VRFB, the LSMO/LCMO VRFB and pristine VRFB were tested for 500 cycles. As shown in Fig. S22, the LSMO/LCMO VRFB runs for 500 cycles and exhibits a higher discharge capacity, while the pristine VRFB only runs for 126 cycles. Moreover, as the efficiency of two VRFBs shown in Fig. 5h, EE and VE of the LSMO/LCMO VRFB are higher than those of the pristine VRFB, the initial EE of LSMO/LCMO VRFB is 76.6%, 17.6% higher than that of the pristine VRFB, and the capacity decay rate of LSMO/LCMO VRFB in 500 cycles is only 0.0216%. Besides, LSMO/LCMO VRFB presents a lower system resistance than the pristine VRFB (Fig. S23). Overall, within the category of metal oxide and bifunctional catalysts, the developed catalyst demonstrates a meaningful advantage at comprehensive performance against other state-of-the-art counterparts (Table S3).

## Conclusion

In summary, this work demonstrates a bidirectional electronic structure engineering strategy for enhancing the catalytic activity of LaMnO_3_ perovskite toward vanadium redox reactions. Through rational doping with Sr and Ce, we have achieved targeted modulation of Mn chemical environment and oxygen defect chemistry. Sr doping promotes the formation of Mn^4+^ and establishes efficient electron delocalization pathways via the double-exchange mechanism, accelerating the charge transfer kinetics for V^3+^/V^2+^ redox couple. In parallel, Ce doping induces oxygen vacancy formation and increases Mn^3+^, creating favorable sites for vanadium ion adsorption and electron transfer in VO^2+^/VO_2_^+^ reaction. Theoretical calculation corroborates that the modified electronic structures in doped LaMnO_3_ enhance both the adsorption affinity and charge transfer efficiency for vanadium ion. When implemented as composite electrodes in practical VRFB, LSMO-20 and LCMO-10 catalysts enable exceptional performance improvements, achieving a 17% enhancement in EE at 150 mA cm^−2^ and maintaining 66.5% EE at 300 mA cm^−2^, where pristine electrodes fail. This study provides fundamental insights into the design of bidirectional electrocatalysts through precise electronic structure control, opening new avenues for developing high-performance materials for flow battery applications.

## Supplementary Information

Below is the link to the electronic supplementary material.

Supplementary file1 (DOCX 4285 KB)
