# Supplementary material for: Bidirectionally Enhanced Reaction Kinetics in Vanadium Redox Flow Battery via Regulating Mixed-Valence States in Perovskite Electrodes
Source: Nanomicro Lett. 2026 Feb 3;18:233. doi: 10.1007/s40820-025-02060-0 (PMC12868327; doi:10.1007/s40820-025-02060-0)
Supplement: Supplementary file 1 — Supplementary file1 (DOCX 4285 KB) [file 40820_2025_2060_MOESM1_ESM.docx]

Supporting Information for

**Bidirectionally Enhanced Reaction Kinetics in Vanadium Redox Flow Battery via Regulating Mixed-Valence States in Perovskite Electrodes**

Yingqiao Jiang^1^, Ming Li^1^, Jiaye Ye^2^ *, Lei Dai^1^, Haoran Jiang^3^ *, Ling Wang^1^, Zhangxing He^1^ *

^1^ School of Chemical Engineering, North China University of Science and Technology, Tangshan 063009, P. R. China

^2^ School of Chemistry and Physics, Faculty of Science, Queensland University of Technology, Brisbane, QLD 4000, Australia

^3^ Department of Energy and Power Engineering, Tianjin University, Tianjin 300072, P. R. China

*Corresponding authors. E-mail: [zxhe@ncst.edu.cn](mailto:zxhe@ncst.edu.cn) (Zhangxing He); [jiaye.ye@qut.edu.au](mailto:jiaye.ye@qut.edu.au) (Jiaye Ye); [jianghaoran@tju.edu.cn](mailto:jianghaoran@tju.edu.cn) (Haoran Jiang)

**Supplementary Figures and Tables**

**
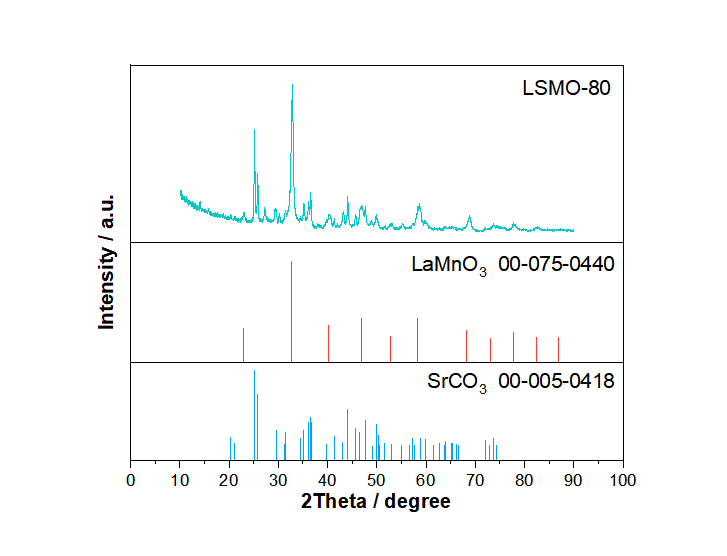
**

**Fig. S1** The XRD patterns of LSMO-80

The effect of Sr doping on the morphology of LaMnO_3_ perovskite was observed by scanning electron microscope (SEM), as shown in **Fig. S2.** In **Fig**. **S2a, b**, it can be seen that the undoped LaMnO_3_ perovskite was composed of small spherical nanoparticles, and the particle size was evenly dispersed. Between 50~100 nm, there was no obvious agglomeration. **Fig**. **S2c-h** is the SEM image of LaMnO_3_ perovskite samples with different Sr doping ratios. Compared with LMO and LSMO perovskite samples, it was found that Sr doping reduces the size of LaMnO_3_ nanoparticles, which was related to the decrease of LaMnO_3_ lattice size by Sr doping. In addition, Sr-doped LaMnO_3_ perovskites still maintain good dispersibility.


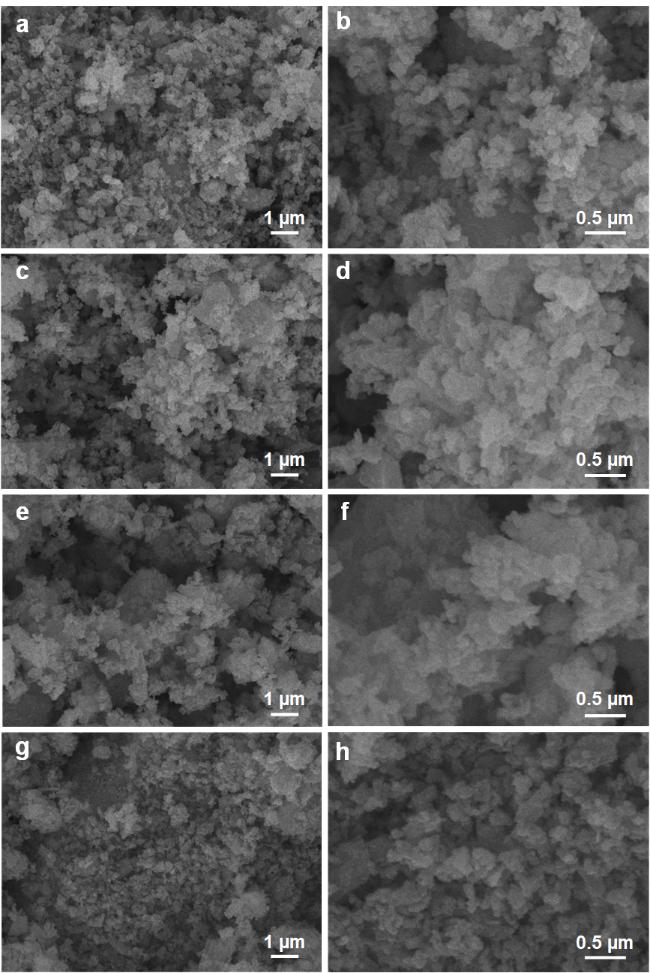


**Fig**. **S2** SEM images of LMO (**a, b**), LSMO-10 (**c, d**), LSMO-20 (**d, e**) and LSMO-40 (**g, h**)

**Figure** **S3** shows the SEM images of LaMnO_3_ perovskite samples with different Ce doping ratios. It can be seen that the Ce-doped LaMnO_3_ perovskite samples are composed of nanoparticles, and the particle size distribution is in the range of 50-100 nm. The particle size is uniformly dispersed without obvious agglomeration, indicating that Ce doping does not affect the dispersion of LaMnO_3_ perovskite. Further comparison of LMO and LCMO samples shows that Ce has no significant effect on the nanoparticle size of LaMnO_3_ perovskite.


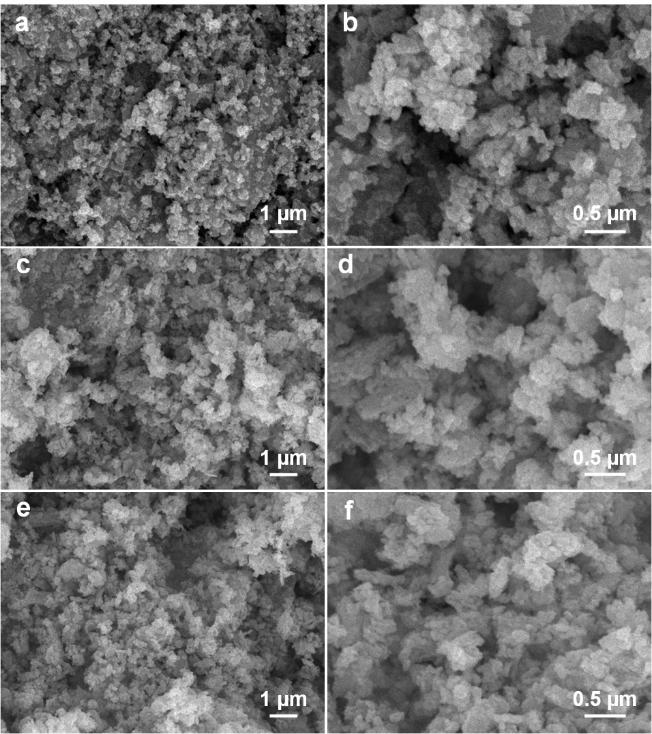


**Fig**. **S3** SEM images of LCMO-05 (**a, b**), LCMO-10 (**c, d**) and LCMO-15 (**e, f**)


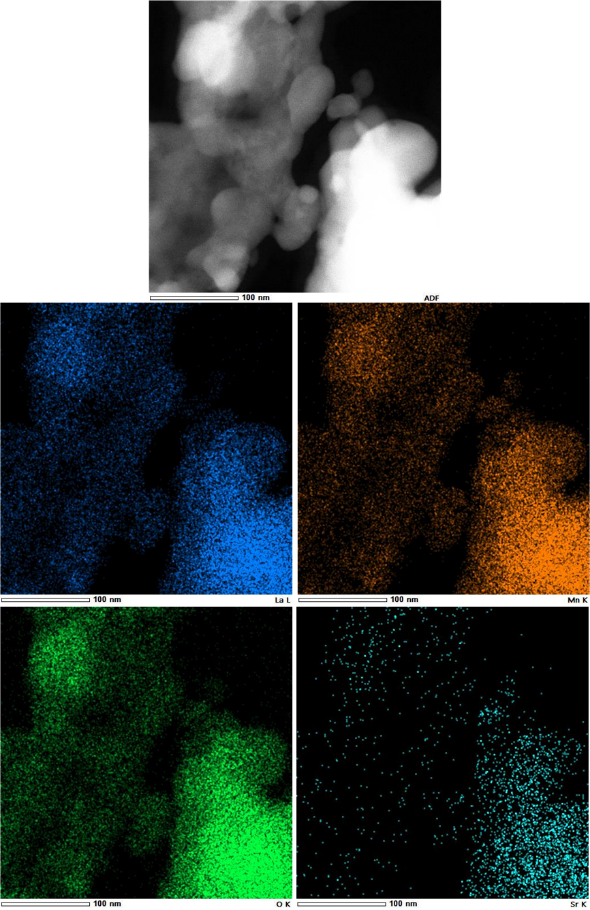


**Fig**. **S4** TEM and the corresponding EDS mapping images of LSMO-20


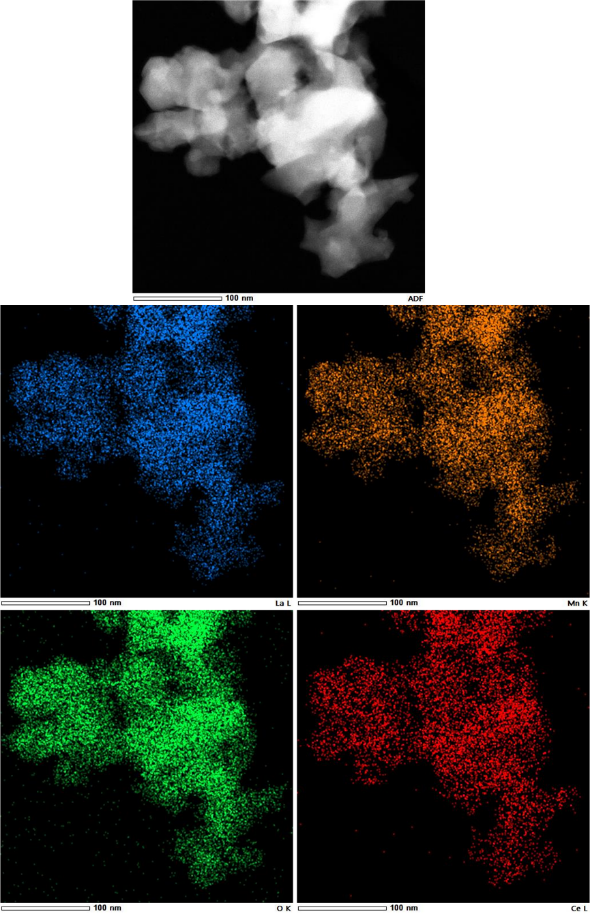


**Fig**. **S5** TEM and the corresponding EDS mapping images of LCMO-10

The chemical elemental compositions of the surfaces of the LMO, LSMO, and LCMO samples were semi-quantitatively analyzed using X-ray photoelectron spectroscopy (XPS), as shown in **Figs**. **S6** and **S7**. In **Fig**. **S6**, it is observed that the energy spectrum of LMO exhibits characteristic peaks for La 3d (866.3 and 863.4 eV), Mn 2p (642.0 and 653.8 eV), O 1s (529.4 eV), and C 1s (284.8 eV). This indicates that the LMO sample consist of four chemical elements of C, La, Mn, and O. The presence of element C is attributed to surface contamination of the sample, which can be used for energy spectrum standard peak correction. In addition to these four elements, the characteristic peaks of Sr 3d and Ce 3d appeared at 132.86 and 898.98 eV, respectively, in all LSMO (**Fig**. **S6**) and LCMO (**Fig**. **S7**) samples. This suggests that Sr and Ce were successfully introduced into LaMnO_3_, and the intensities of the characteristic peaks of Sr 3d and Ce 3d increased continuously with the increase of the doping ratios of Sr and Ce elements. Characteristic peak intensities are increasing.

**Fig. S6** XPS survey spectra of LMO and LSMO samples

**Fig. S7** XPS survey spectra of LMO and LCMO samples

**Figure S8** shows the XPS spectra of Sr of LSMO-10, LSMO-20, and LSMO-40 samples. There are two peaks corresponding to the binding energies of Sr 3d_3/2_ (134.15 eV) and Sr 3d_3/2_ (132.39 eV), respectively, with the spacing of the two peaks of 1.76 eV, suggesting Sr^2+^ doped into LaMnO_3_ lattice. The peak intensity of the XPS energy spectrum of Sr 3d increases with the increase in the doped Sr content, but the position of the double peaks remains unchanged.

**Fig. S8** XPS spectra of Sr 3d of LSMO samples

**Fig. S9** XPS spectra of Ce 3d of LCMO-05, LCMO-10, and LCMO-15 samples


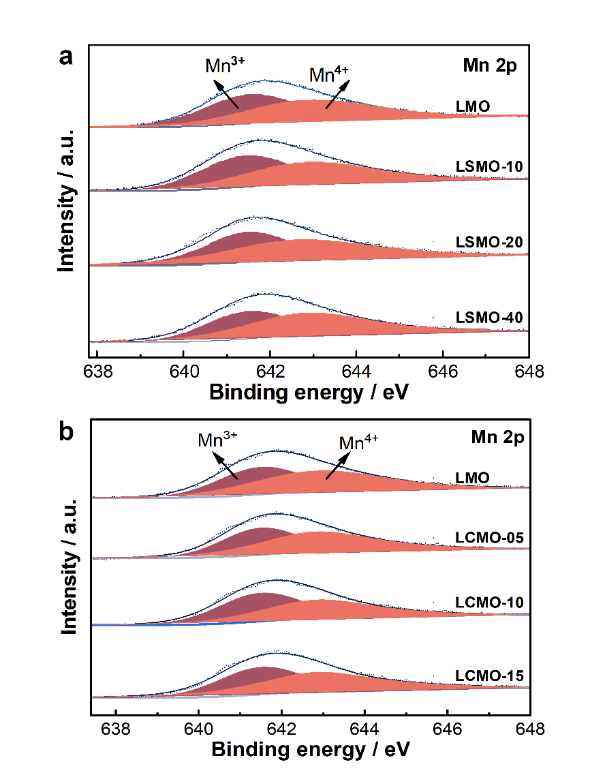

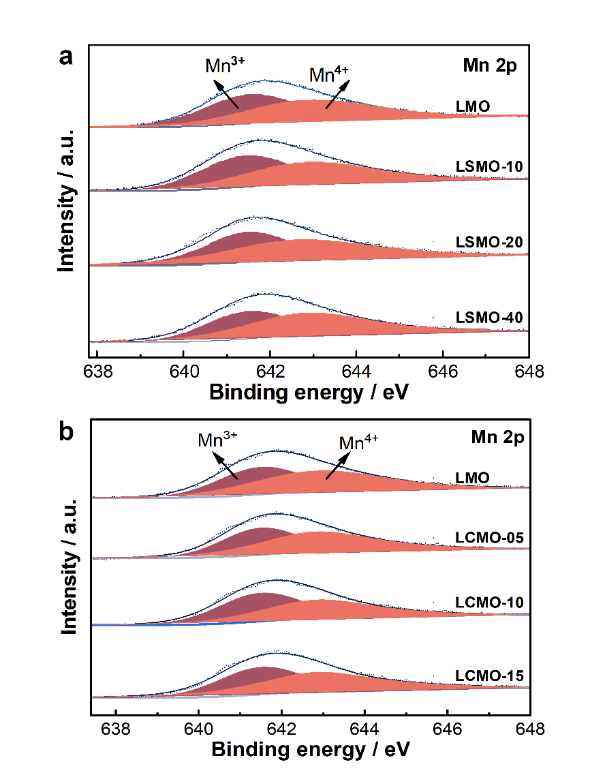


**Fig. S10** XPS spectra of Mn 2p of LSMO (**a**) and LCMO (**b**) samples

In addition, with the increase in the Sr and Ce contents, the concentration of surface O_ads_ in LSMO and LCMO samples first increases and then decreases. In LSMO and LCMO samples, when the doping ratio of Sr and Ce reaches 20% and 10%, respectively, the concentration of O_ads_ in the samples reaches its highest level. Additionally, the structural defects increase, which in turn provides more active sites for vanadium redox reactions.


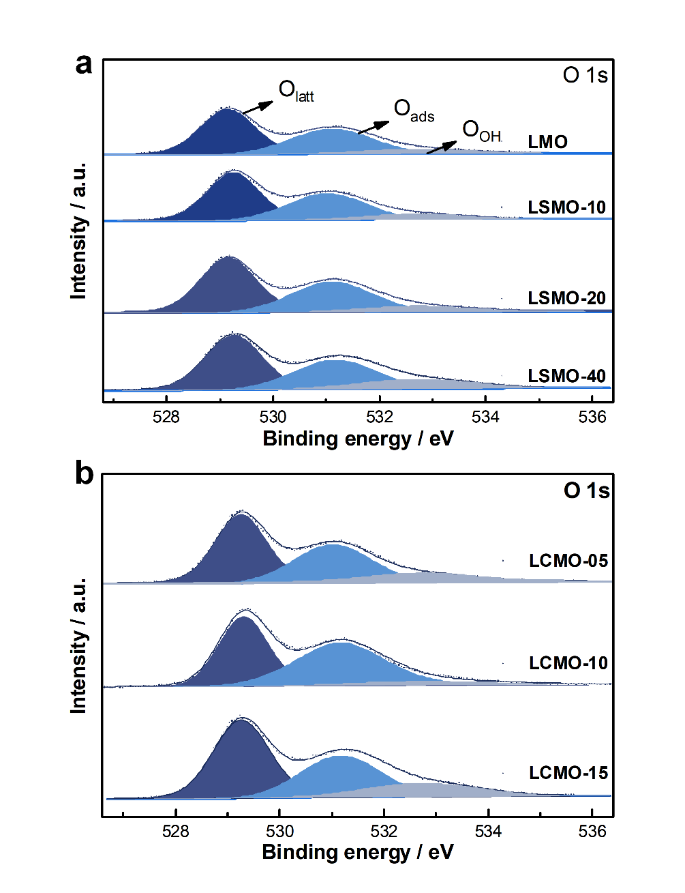


**Fig. S11** XPS spectra of O ls of LSMO (**a**) and LCMO (**b**) catalysts


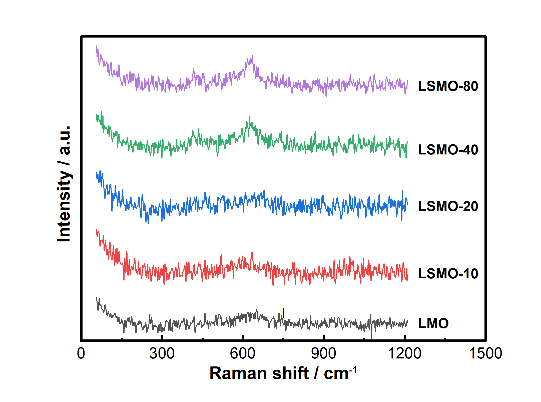


**Fig. S12** Raman spectra of LSMO


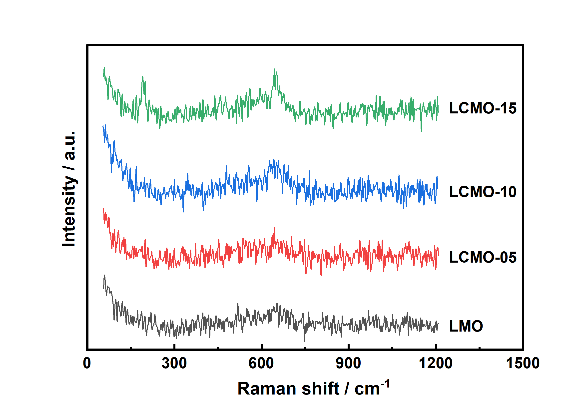


**Fig. S13** Raman spectra of LCMO


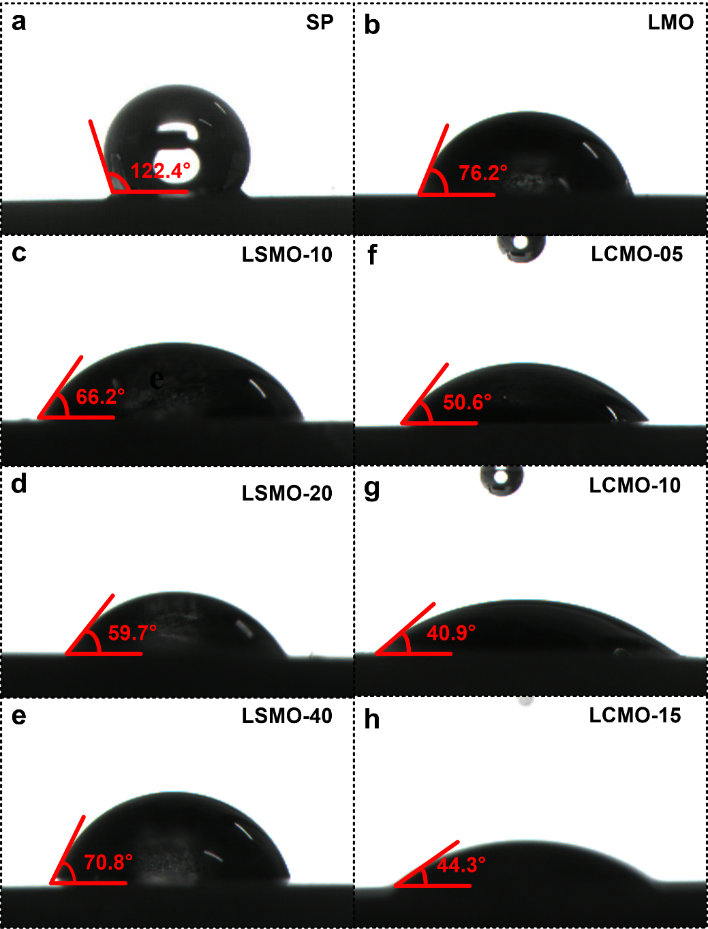


**Fig. S14** The water contact angle of SP (**a**), LMO (**b**), LSMO (**c-e**) and LCMO (**f-h**) samples

To explore the effect of Sr-doped LaMnO_3_ perovskite on the V^3+^/V^2+^ redox reaction polarization and mass transfer process, the CVs of LMO and LSMO electrodes were tested with different scan rates, as shown in **Fig. S15**. It can be observed that the redox peak current density of the electrodes increased continuously with the increase of the scan rate. At the same time, the redox peak potential difference increased continuously due to the increase of the polarization of the electrode reaction. Comparing the multi-scan rate CV curves of the four groups of electrodes, the LMO electrode shows the smallest increase in the redox peak current density and the largest change in the peak potential difference. Meanwhile, the LMO electrode undergoes an increasingly severe hydrogen precipitation side reaction. The LSMO-10 and LSMO-20 electrodes show good electrochemical stability, and the peak potential difference of LSMO-40 changed greatly. These indicate that Sr doping effectively reduces the polarization and side reaction of the V^3+^/V^2+^ redox reaction.


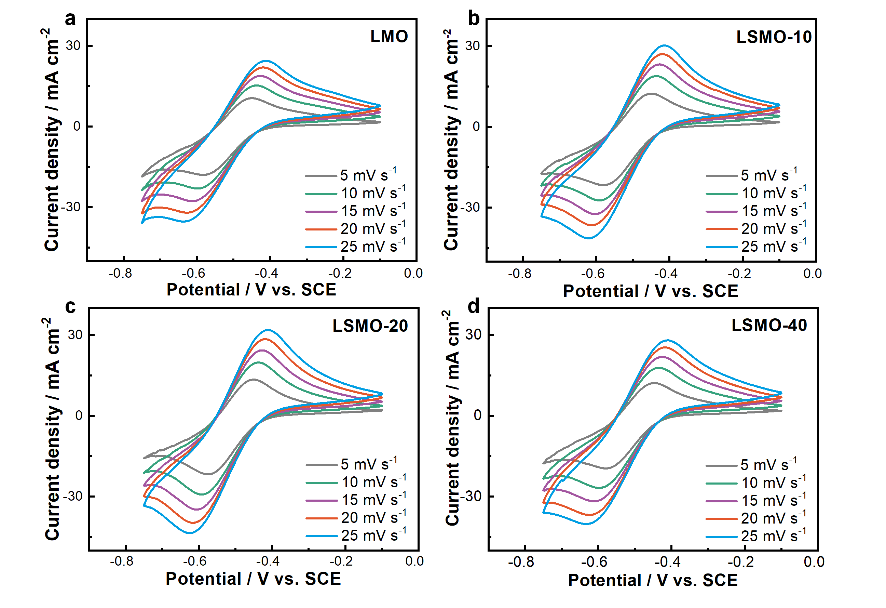


**Fig. S15** The CV curves of LMO and LSMO electrodes at different scan rates in 1.6 M V^3+^ + 3.0 M H_2_SO_3_ solution

**Table S1** Corresponding fitting electrochemical parameters obtained from the fitting data in **Fig. 3c** for LMO and LSMO electrodes based on the equivalent circuit in **Fig. 3g**

| Electrode | R_s_ / Ω | Q_t_ | | R_ct_ / Ω | Q_m_ | |
| --- | --- | --- | --- | --- | --- | --- |
|  |  | Y_0,1_ | n_1_ |  | Y_0,2_ | n_2_ |
| LMO | 10.54 | 1.76×10^–2^ | 0.70 | 15.76 | 2.75×10^–5^ | 0.77 |
| LSMO-10 | 10.32 | 2.68×10^–2^ | 0.67 | 8.02 | 3.85×10^–5^ | 0.84 |
| LSMO-20 | 9.66 | 3.62×10^–2^ | 0.80 | 6.18 | 5.30×10^–5^ | 0.80 |
| LSMO-40 | 10.57 | 2.60×10^–2^ | 0.55 | 9.15 | 2.81×10^–5^ | 0.87 |

In order to investigate the effect of Ce-doped LaMnO_3_ perovskites on the electrochemical stability and mass-transfer processes of the VO^2+^/VO^2+^ redox reaction, CV tests were performed on LMO and LCMO electrodes with different sweep speeds. As shown in **Fig. S16**, it can be observed that as the scan rate increases, redox peak potential difference increase due to increasing polarisation at the electrode reaction interface. Comparing the multi-scan rate CV curves of the 4 sets of electrodes, LCMO-10 has the fastest increase rate in peak current density and the smallest increase extent in peak potential difference. It indicates that Ce doping improves the electrochemical kinetics of the electrode and reduces the electrode reaction polarisation.


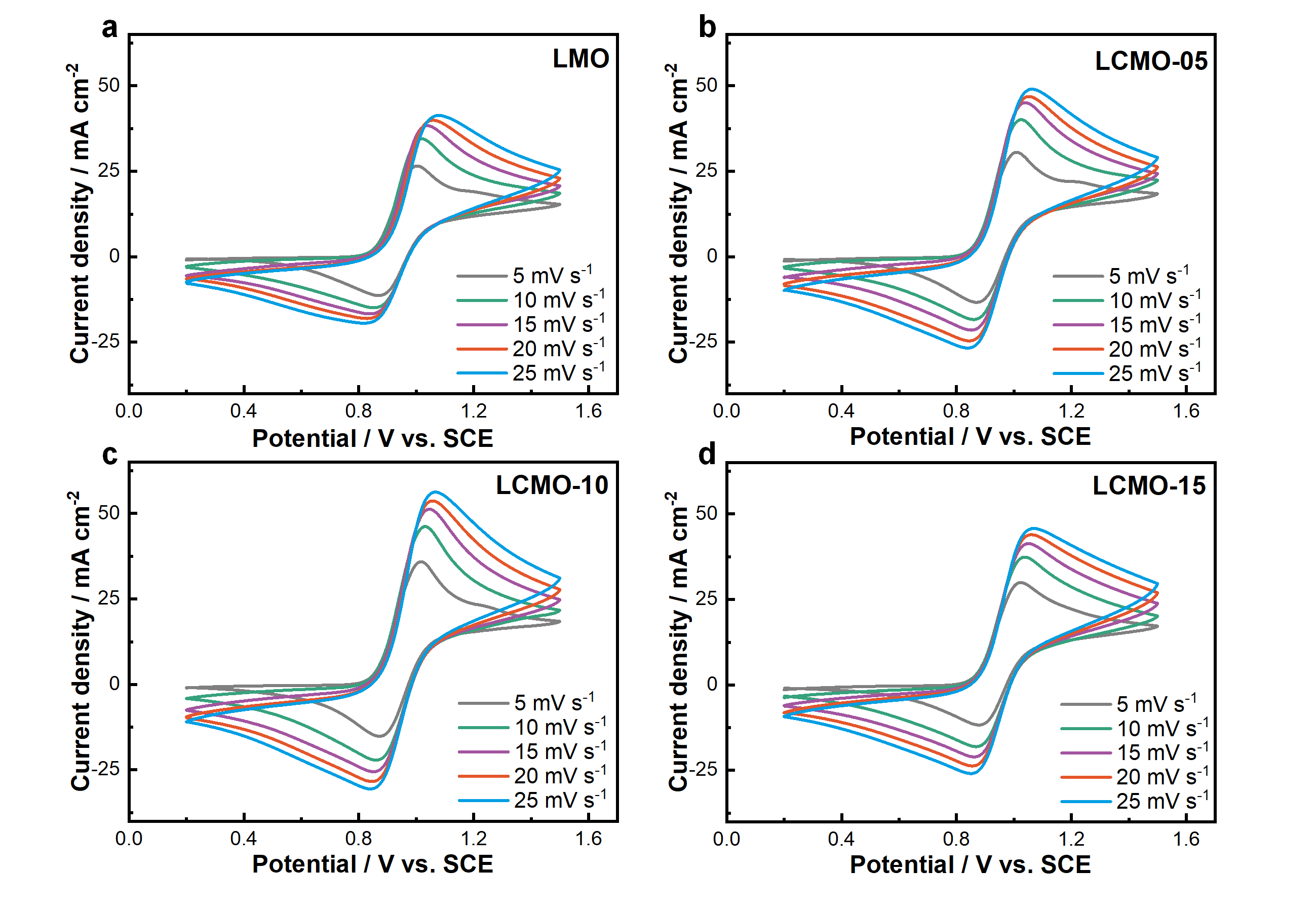


**Fig. S16** The CV curves of LMO and LCMO electrodes at different scan rates in 1.6 M V^3+^ + 3.0 M H_2_SO_3_ solution


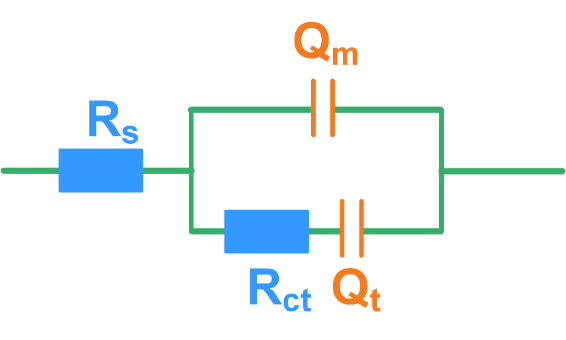


**Fig. S17** The fitting equivalent circuit for the Nyquist plots in **Fig. 3c, f**

**Table S2** Corresponding fitting electrochemical parameters obtained from the fitting data in **Fig. 3c** for LMO and LSMO electrodes based on the equivalent circuit in **Fig. 3g**

| Electrode | R_s_ / Ω | Q_t_ | | R_ct_ / Ω | Q_m_ | |
| --- | --- | --- | --- | --- | --- | --- |
|  |  | Y_0,1_ | n_1_ |  | Y_0,2_ | n_2_ |
| LMO | 10.54 | 1.76×10^–2^ | 0.70 | 15.76 | 2.75×10^–5^ | 0.77 |
| LSMO-10 | 10.32 | 2.68×10^–2^ | 0.67 | 8.02 | 3.85×10^–5^ | 0.84 |
| LSMO-20 | 9.66 | 3.62×10^–2^ | 0.80 | 6.18 | 5.30×10^–5^ | 0.80 |
| LSMO-40 | 10.57 | 2.60×10^–2^ | 0.55 | 9.15 | 2.81×10^–5^ | 0.87 |

**Table S2** The corresponding fitting electrochemical parameters obtained from the fitting data in **Fig. 3f** for LMO and LCMO electrodes based on the equivalent circuit in **Fig. 3g**

| Electrode | R_s_ / Ω | Q_t_ | | R_ct_ / Ω | Q_m_ | |
| --- | --- | --- | --- | --- | --- | --- |
|  |  | Y_0,1_ | n_1_ |  | Y_0,2_ | n_2_ |
| LMO | 7.09 | 1.20×10^–2^ | 0.53 | 35.02 | 2.14×10^–5^ | 0.85 |
| LCMO-05 | 7.41 | 1.34×10^–2^ | 0.59 | 20.39 | 3.22×10**^-^**^5^ | 0.85 |
| LCMO-10 | 8.23 | 2.00×10^–2^ | 0.48 | 12.72 | 3.60×10^–5^ | 0.92 |
| LCMO-15 | 8.71 | 1.35×10^–2^ | 0.51 | 24.09 | 2.69×10**^-^**^5^ | 0.88 |


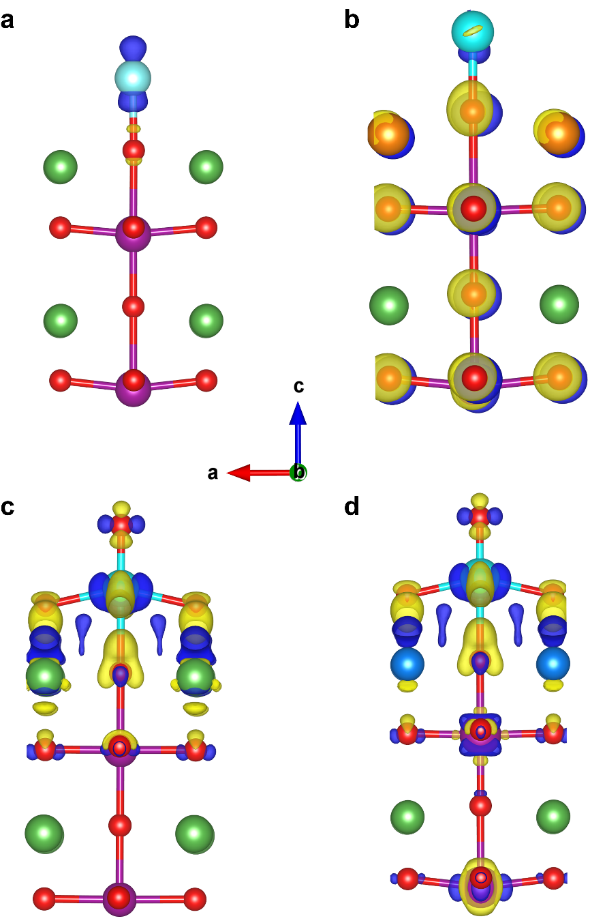


**Fig. S18** The left view of charge difference analysis for V^3+^-LaMnO_3_ (**a**), V^3+^-Sr doped LaMnO_3_ (**b**), VO^2+^-LaMnO_3_ (**c**), VO^2+^-Ce doped LaMnO_3_ (**d**)


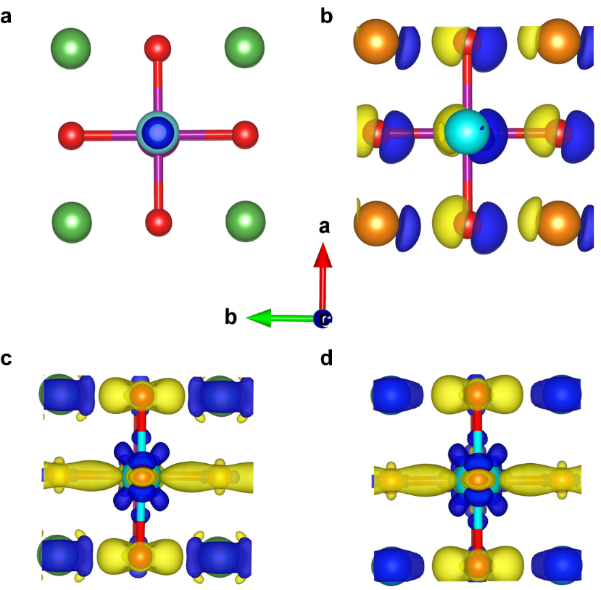


**Fig. S19** The top view of charge difference analysis for V^3+^-LaMnO_3_ (**a**), V^3+^-Sr doped LaMnO_3_ (**b**), VO^2+^-LaMnO_3_ (**c**), VO^2+^-Ce doped LaMnO_3_ (**d**)


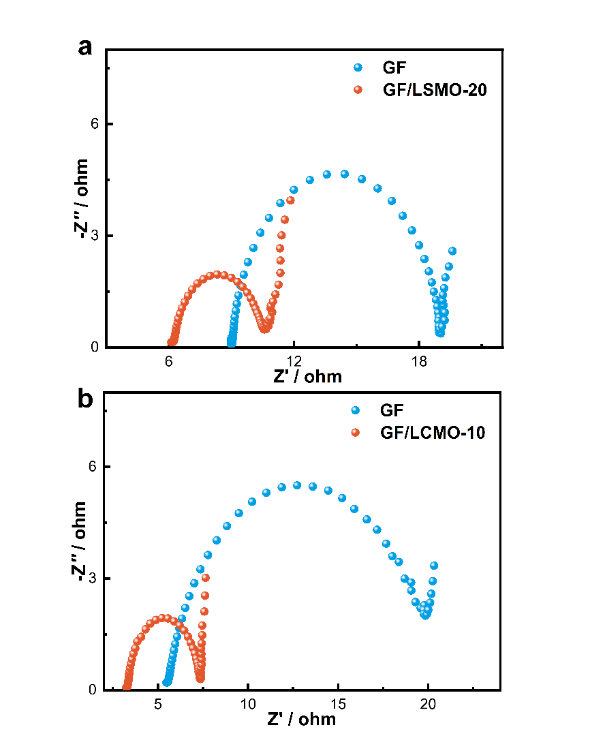


**Fig. S20** The Nyquist plots of GF electodes for V^3+^/V^2+^ (**a**) and VO^2+^/VO_2_^+^ (**b**) redox reaction at polarization potentical

**Figure S21** shows the charge-discharge curves of the two batteries at different current densities. It can be seen that with the increase of current density, the polarisation of the two groups of batteries intensifies, the overvoltage increases continuously, the spacing between the charging and discharging platforms increases continuously, and the charging and discharging curves of the modified VRFB are more stable compared with those of the pristine VRFB. Meanwhile, at the same current density, the modified VRFB has a lower charging platform and a higher discharge platform, and at 150 mA cm^-2^, the onset voltage of charging of the modified VRFB decreases by 0.15 V, and the onset voltage of discharging increases by 0.29 V. The LSMO-20 and the LCMO-10 jointly reduce the polarisation of the VRFB and improve the energy density of the VRFB.

**
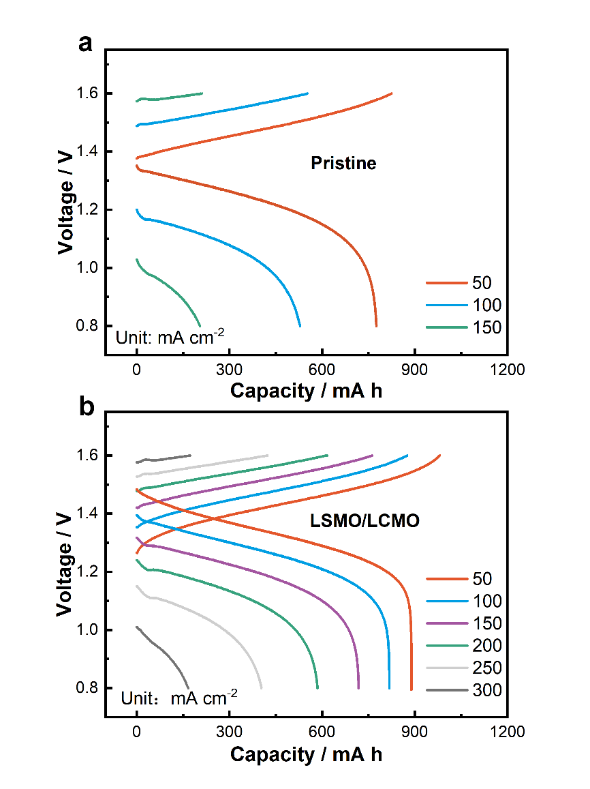
**

**Fig**. **S21** The chairge and discharge curves of the pristine (**a**) and modified batteries (**b**) at different current densities
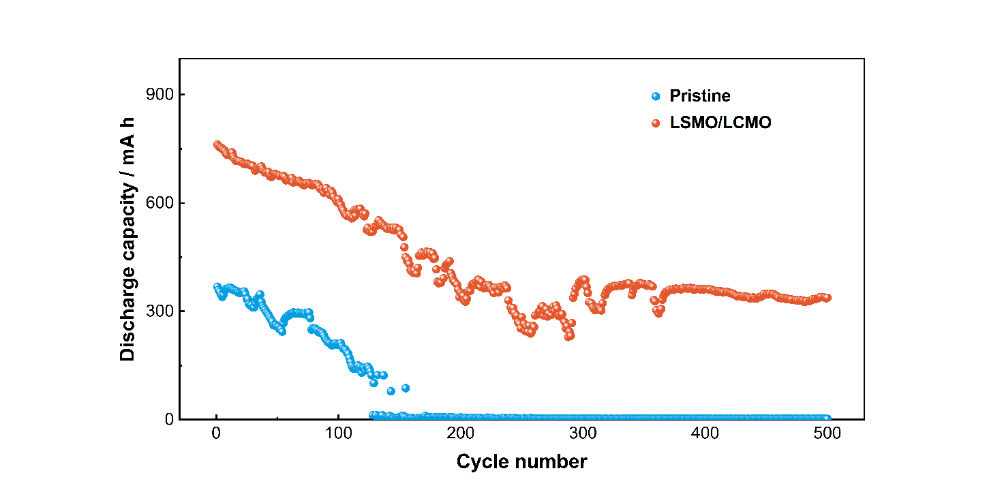


**Fig**. **S22** The discharge capacity of the pristine and modified batteries at 150 mA cm^-2^ for 500 cycles

**
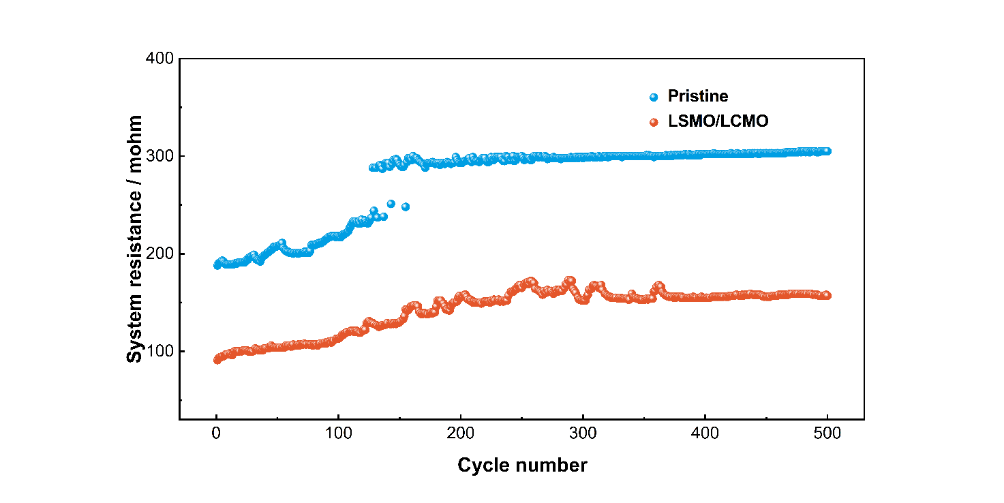
**

**Fig**. **S23** The system resistance of the pristine and modifed bateries at 150 mA cm^−2^ for 500 cycles

**Table S3** Performance comparison of LSMO/LCMO with representative VRFB catalyst materials

| Material type | Catalyst material | Cathode/Anode | VRFB performance | | long-term cycling performance | | Refs. |  |
| --- | --- | --- | --- | --- | --- | --- | --- | --- |
|  |  |  | Max current density  (mA cm^-2^) | EE | Cycle Stability | Lifetime |  |  |
| Carbon | 1C nano layer | Cathode/Anode | 250 | 72% | 150 | 450 | [S1] |  |
|  | Lignin | Cathode | 250 | 75% | 100 | 1000 | [S2] | |
|  | C–TA/TAEA | Cathode/Anode | 500 | 56% | 400 | 1000 | [S3] | |
|  | N, P co-doped carbon fiber | Cathode/Anode | 200 | 60% | 100 | 150 | [S4] | |
|  | GFD-Zn-H | Cathode/Anode | 500 | 31% | - | - | [S5] | |
|  | DB-GF | Cathode/Anode | 300 | 73% | 300 | 300 | [S6] | |
|  | SDG-PC | Cathode/Anode | 500 | ~56% | 400 | 1000 | [S7] | |
|  | POGC | Cathode | 250 | ~58% | 200 | 1000 | [S8] | |
|  | N/Co,Cu@GF | Cathode | 350 | 66% | 200 | 350 | [S9] | |
|  | SGTA/  TiO_2_ | Anode | 325 | 54% | 100 | 100 | [S10] | |
| Metal oxide | Cu-Co_3_O_4_ | Cathode | 200 | 76.4% | 200 | 300 | [S11] | |
|  | V_A_-Mn_3_O_4_ | Anode | 450 | ~68% | 300 | 1000 | [S12] | |
|  | Bi_2_WO_6_ | Anode | 250 | 89.7% | 250 | 1000 | [S13] | |
|  | VNbMoTaWO_x_ | Cathode | 120 | 77.2% | 100 | 250 | [S14] | |
|  | medium-entropy oxide | Cathode | 350 | ~63% | 200 | 500 | [S15] | |
|  | TiO_2_ | Anode | 400 | ~72% | 200 | 600 | [S16] | |
|  | NiMoO_4_ | Cathode/Anode | 200 | ~63% | 100 | 100 | [S17] | |
|  | SrZrO_3_ | Cathode/Anode | 250 | ~63% | 150 | 500 | [S18] | |
|  | WO_3_ | Anode | 300 | 73.6% | 250 | 900 | [S19] | |
|  | NdFeO_3_ | Cathode | 250 | 65% | 150 | 1000 | [S20] | |
| Other | Ti_3_C_2_T_x_-N/m | Anode | 450 | 64% | 150 | 500 | [S21] | |
|  | IL-MW-MXene | Anode | 200 | 83.6% | 100 | 200 | [S22] | |
|  | Nb_2_CT_x_/Nb_2_O_5_ | Cathode/Anode | 400 | 65% | 150 | 800 | [S23] | |
|  | Mo_2_C−Mo_2_N | Cathode | 450 | ~67% | 300 | 1000 | [S24] | |
|  | This work | Cathode/Anode | 300 | 67% | 150 | 500 |  | |

**Supplementary References**

1. Y. Yang, X. Wang, Y. Wang, G. Qiu, Z. Song et al., Functional nano-carbon layer decorated carbon felt electrode for vanadium redox flow batteries. J. Energy Chem. **106**, 735–741 (2025). <https://doi.org/10.1016/j.jechem.2025.02.056>
2. X. He, L. Li, S. Yan, H. Fu, F. Zhong et al., Advanced electrode enabled by lignin-derived carbon for high-performance vanadium redox flow battery. J. Colloid Interface Sci. **653**, 1455–1463 (2024). <https://doi.org/10.1016/j.jcis.2023.10.005>
3. H. An, S. Jeon, Y. Chung, Sustainable synthesis of edge-dominant pyridinic nitrogen-doped carbon from tannic acid for high-performance vanadium redox flow batteries. Energy Environmental Mater. e70033 (2025). <https://doi.org/10.1002/eem2.70033>
4. X. Chen, C. Wu, Y. Lv, S. Zhang, Y. Jiang et al., Highly active nitrogen-phosphorus Co-doped carbon fiber@graphite felt electrode for high-performance vanadium redox flow battery. J. Colloid Interface Sci. **677**(Pt B), 683–691 (2025). <https://doi.org/10.1016/j.jcis.2024.08.091>
5. D.R. Lobato-Peralta, A.J. Molina-Serrano, J.M. Luque-Centeno, B. Sánchez-Laganga, D. Sebastián et al., Improving energy storage properties of carbon felt electrodes for vanadium redox flow batteries *via* ZIF modifications. Chem. Eng. J. **515**, 163534 (2025). <https://doi.org/10.1016/j.cej.2025.163534>
6. Q. Wang, X. Shan, H. Liu, L. Chen, J. Cao et al., Interfacial copolymerization electrode enhanced high performance vanadium redox flow batteries. Chem. Eng. J. **524**, 169779 (2025). <https://doi.org/10.1016/j.cej.2025.169779>
7. H. An, S. Jeon, J. Park, Y. Chung, Microwave and NaCl driven synthesis of P-doped graphitic carbon at atmospheric pressure for long-life vanadium redox flow batteries. J. Mater. Chem. A **13**(19), 13924–13934 (2025). <https://doi.org/10.1039/D4TA09283J>
8. S. Jeon, H. An, Y. Chung, Microwave-assisted synthesis of P-doped and O-rich graphitic carbon catalyst for vanadium redox flow batteries. Chem. Eng. J. **478**, 147198 (2023). <https://doi.org/10.1016/j.cej.2023.147198>
9. X. Cheng, Z. Wang, L. Xia, J. Zhang, Y. Min et al., MOF-derived nitrogen, sulfur, cobalt, and copper Co-doped graphite felt for high-efficiency vanadium redox flow battery electrodes. J. Colloid Interface Sci. **687**, 1–13 (2025). <https://doi.org/10.1016/j.jcis.2025.02.041>
10. M.K. Mutuma, H. Jung, Acetylacetonate-modified TiO_2_ nanoparticles coated on the carbon felt as the negative electrode of vanadium redox flow battery for reducing HER and enhancing V(3+)/V(2+) redox reactions. J. Colloid Interface Sci. **679**(Pt B), 155–164 (2025). <https://doi.org/10.1016/j.jcis.2024.10.088>
11. A.M. Demeku, C.-H. Guo, D.M. Kabtamu, Z.-J. Huang, G.-C. Chen et al., Enhanced electrochemical performance of copper-doped cobalt oxide nanowire-modified graphite felt as positive electrode material for vanadium redox flow batteries. Chem. Eng. J. **505**, 159170 (2025). <https://doi.org/10.1016/j.cej.2024.159170>
12. X. Zhang, K. Ao, J. Shi, X. Yue, A. Valencia et al., The critical role of atomic-scale polarization in transition metal oxides on vanadium-redox electrochemistry. Adv. Mater. **37**(13), e2420510 (2025). <https://doi.org/10.1002/adma.202420510>
13. M.M. Omran, T. Al Najjar, N.K. Allam, E.N. El Sawy, Carbon felt coated with tungsten–bismuth-based oxides as highly active and selective negative electrodes for high power density all-vanadium redox flow batteries. J. Mater. Chem. A **13**(27), 21707–21724 (2025). <https://doi.org/10.1039/D5TA00882D>
14. K. Tiwari, C.-H. Wang, B.-S. Lou, C.-J. Wang, I. Moirangthem et al., High entropy alloy oxide coating of VNbMoTaWOx as a novel electrode modification of vanadium redox flow batteries. J. Energy Storage **94**, 112344 (2024). <https://doi.org/10.1016/j.est.2024.112344>
15. X. Pan, X. Cheng, T. Deng, L. Xia, J. Zhang et al., MOF-derived high-entropy oxide-modified graphite felt for enhanced electrochemical performance in vanadium redox flow batteries. J. Mater. Sci. Technol. **247**, 44–54 (2026). <https://doi.org/10.1016/j.jmst.2025.05.033>
16. R. Huang, S. Su, Y. Wang, S. Liu, Z. He et al., Nature of oxygen vacancy in accelerating redox kinetics of V(2+)/V(3+) in flow batteries. J. Colloid Interface Sci. **690**, 137281 (2025). <https://doi.org/10.1016/j.jcis.2025.137281>
17. M.-X. Zhai, X.-R. Chen, T.-K. Cheng, Y.-Q. Jiang, L. Wang et al., NiMoO_4_nanorods with rich catalytic sites in situ-modified graphite felt composite electrode for vanadium redox flow battery. Rare Met. **44**(8), 5383–5392 (2025). <https://doi.org/10.1007/s12598-025-03254-6>
18. S. Zhang, L. Ma, Y. Wu, M. Xie, W. Liu et al., Constructing structural defects on perovskite surface to accelerate electrode kinetics for vanadium redox flow batteries. Small **21**(36), e06245 (2025). <https://doi.org/10.1002/smll.202506245>
19. R. Huang, S. Liu, Z. He, G. Ye, W. Zhu et al., The role of proton in high power density vanadium redox flow batteries. ACS Nano **17**(19), 19098–19108 (2023). <https://doi.org/10.1021/acsnano.3c05037>
20. R. Mu, W. Zhang, Z. Chang, C. Zhang, B. Wang et al., Unlocking high-efficiency energy storage: neodymium ferrite perovskite as a cathode catalyst for vanadium redox flow batteries. J. Mater. Chem. A **13**(41), 35836–35846 (2025). <https://doi.org/10.1039/D5TA06257H>
21. X. Chen, L. Li, Y. Jiang, Z. Feng, Q. Li et al., Manipulating the local electronic structure microenvironment at the MXene interface to achieve efficient anode for vanadium redox flow battery. J. Energy Chem. **104**, 118–126 (2025). <https://doi.org/10.1016/j.jechem.2024.11.062>
22. M. Jing, X. Li, H. Yu, X. An, Z. Liu et al., Ionic liquid etched and microwave-assisted delaminated MXene as an excellent electrocatalyst for the hysteretic negative reaction of vanadium redox flow batteries. Chem. Eng. J. **455**, 140789 (2023). <https://doi.org/10.1016/j.cej.2022.140789>
23. T. Yuan, X. Chen, Y. Zhao, Y. Jiang, B. Li et al., Bridging the charge gap: Nb2CT x/Nb_2_O_5_ Schottky heterojunctions as electronic highways in vanadium redox flow battery. J. Energy Chem. **113**, 771–779 (2026). <https://doi.org/10.1016/j.jechem.2025.09.064>
24. X. Zhang, X. Ye, A. Valencia, F. Liu, K. Ao et al., Asymmetric chemical potential activated nanointerfacial electric field for efficient vanadium redox flow batteries. ACS Nano **17**(21), 21799–21812 (2023). <https://doi.org/10.1021/acsnano.3c07732>
